# Natural Deep Eutectic Solvent–Dipotassium Phosphate Aqueous Two-Phase Systems: Physicochemical Characterization, Selective Partitioning of Amino Acids and Glucose, and Functional Insight into Maillard Reaction Applications

**DOI:** 10.1021/acssuschemeng.5c03053

**Published:** 2025-07-21

**Authors:** Kangni Chen, Antonio Dario Troise, Anton Bunschoten, Sabrina De Pascale, Andrea Scaloni, Vincenzo Fogliano, Ashkan Madadlou

**Affiliations:** † Food Quality and Design Group, 4508 Wageningen University & Research, 6708WG Wageningen, The Netherlands; ‡ Proteomics, Metabolomics & Mass Spectrometry Laboratory, 9327 ISPAAM, National Research Council, 80055 Portici, Italy; § BioNanoTechnology, Wageningen University and Research, 6708WG Wageningen, The Netherlands; ∥ School of Food and Nutritional Sciences, University College Cork (UCC), Cork T12 Y337, Ireland

**Keywords:** hydrogen bond acceptors, hydrogen bond donors, partition, water content, water-in-water, Maillard reaction

## Abstract

Despite the growing interest in natural deep eutectic solvents (NADESs) for green separation, critical aspects of their structural stability in aqueous two-phase systems (ATPS), solute partitioning mechanisms, and potential as reaction media remain poorly understood. This study investigates the development and application of NADES-K_2_HPO_4_ ATPS. Four NADES formulations, namely, betaine-glycerol (Bet:Gly), betaine-propylene glycol (Bet:PG), choline chloride-glycerol (ChCl:Gly), and choline chloride-propylene glycol (ChCl:PG), were synthesized and characterized using ^1^H NMR and differential scanning calorimetry (DSC). The phase-forming ability of the NADES-K_2_HPO_4_ ATPS was influenced by the hydrophobicity of the NADES; specifically, the Bet:PG formulation required the lowest K_2_HPO_4_ concentration (25.1 wt %) for phase separation. In these systems, the hydrophobic NADES-rich phase preferentially partitioned hydrophobic amino acids (e.g., phenylalanine, *K* > 100; alanine, *K* ≈ 10), while glucose was enriched in the K_2_HPO_4_-rich phase (*K* ≈ 0.03). DSC analysis confirmed that the NADESs retained their structural integrity within the ATPSs. The Maillard reactions were performed in Bet:PG-K_2_HPO_4_ ATPSs under strongly alkaline conditions (pH 11.65 in the top phase and 11.34 in the bottom phase) at 37 °C. Results demonstrated that Bet:PG enhances the formation and stabilization of the Amadori compounds through hydrogen-bonding and restricted molecular mobility. Overall, this work demonstrates that NADESs retain their supramolecular structure within ATPSs, enabling their dual functionality as both selective extractants and microreactor media. Specifically, the confined microenvironment enhanced the accumulation and stabilization of Amadori compounds. This suggested that NADES-based ATPSs hold promise as tailored platforms for controlling the reaction pathways.

## Graphical abstract

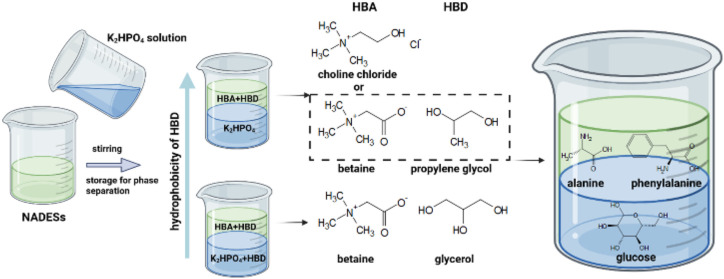

## Introduction

In the field of industrial bioprocessing, the aqueous two-phase system (ATPS) offers an effective method for the isolation and purification of biomolecules such as amino acids, glucose, and enzymes. This system is widely recognized in downstream processing due to its high recovery yield, cost-effectiveness, ease of scaling, and environmental benefits.^1^ ATPSs are formed by segregative phase separation of two water-soluble components: a polymer and a salt, two salts, or two polymers.^2^ A common variation in the formulation of phase-forming components involves the use of natural deep eutectic solvents (NADESs).

NADESs are made from plant metabolites, such as sugars, alcohols, and organic acids. They are known for their exceptional properties, including potential environmental safety, biodegradability, and nontoxicity, making them promising candidates as highly green solvents.^3,4^ NADESs possess physicochemical properties such as adjustable viscosity, low melting point, and high solubilizing capacity across a broad polarity range of compounds.^5^ Their structure consists of hydrogen bond donors (HBDs) and hydrogen bond acceptors (HBAs), which form intermolecular interactions through hydrogen bonding.^6^ The strength of these interactions depends on the molar composition of a NADES. A well-balanced ratio of HBA and HBD results in a clear and homogeneous NADES that remains stable after synthesis.^4^

As phase-forming components in ATPS, NADESs offer significant advantages such as tunable polarity, high solubilizing power, and excellent biocompatibility.^7^ These properties can be exploited to enhance the selectivity and efficiency of biomolecule partitioning, making NADES-based ATPSs particularly promising for green and sustainable bioprocessing. Previous studies have demonstrated the feasibility of forming NADES-salt ATPSs using salts including tripotassium phosphate (K_3_PO_4_), dipotassium phosphate (K_2_HPO_4_), and potassium citrate (K_3_C_6_H_5_O_7_).^8–10^ Among the phosphate salts, K_2_HPO_4_ is notable for its high solubility in water and strong phase-forming ability in ATPSs.^11^ Marappan and co-workers reported its compatibility with diverse NADES formulations such as choline chloride:sorbitol and choline chloride:1,3-propanediol, and its capacity to form stable and reproducible biphasic systems under a wide range of conditions. This reinforces its suitability as a reliable phase-forming component in NADES-based ATPSs.^8^ So far, significant challenges remain in the development of NADES-salt ATPSs. The relationship between the NADES component hydrophobicity and biomolecule partitioning efficiency remains insufficiently quantified. Moreover, the presence of water in ATPSs can significantly influence the structural integrity of the NADESs. Dai et al. demonstrated through Fourier transform infrared (FT-IR) and nuclear magnetic resonance (NMR) analyses that the strong hydrogen-bonding interactions between HBDs and HBAs in NADESs progressively weaken with water dilution.^12^ When the water content reaches approximately 50% (v/v), these interactions are disrupted completely, leading to the breakdown of the NADES superstructure into free individual components. For example, in choline chloride:1,2-propanediol, hydroxyl proton signals in NMR disappeared upon 50% D_2_O addition, indicating the collapse of the hydrogen bond network. This structural disruption alters key physicochemical properties, such as viscosity and solubilizing capacity, and must be considered in ATPS design and application. Also, it remains unclear whether both NADES components can partition into the same phase, which would be necessary to preserve the NADES structure. As an example, Farias, Sosa, Igarashi-Mafra, Coutinho, and Mafra prepared ATPSs composed of K_2_HPO_4_ and a NADES consisting of choline chloride together with fructose, glucose, or sucrose.^13^ Their results indicated that the top phase was rich in HBA (choline chloride), while HBD (sugars) preferentially partitioned into the bottom phase (K_2_HPO_4_-rich phase). Addressing the above-mentioned gaps is essential for optimizing NADES-salt ATPSs for applications such as protein purification and reaction engineering.

NADESs have been reported to facilitate the accumulation of key intermediates in the Maillard reaction, particularly the Amadori compounds. This suggests that NADESs not only act as reaction media but also actively influence the stability and conversion of Maillard precursors and intermediates. Kranz and Hofmann reported that using NADES as a reaction medium significantly enhanced Amadori product yields.^14^ In their study, a malic acid/sucrose-based NADES facilitated the reaction between carnosine and glucose at 100 °C, yielding over 400 μmol/mmol of 1-deoxy-d-fructosyl-*N*-β-alanyl-l-histidine. In contrast, the same reaction conducted in an aqueous solution at 100 °C yielded only ∼10 μmol/mmol, highlighting the superior efficiency of the NADES system. In a following study, Hartl, Frank, Dawid, and Hofmann developed an inert sucrose/sorbitol NADES system and successfully synthesized the taste-modulating compounds *N* ^2^-(1-carboxyethyl)­guanosine 5′-monophosphate (161.8 μmol/mmol) and *N* ^2^-(furfuryl thiomethyl)­guanosine 5′-monophosphate (95.7 μmol/g).^15^ Compared with other NADES systems or aqueous buffered solutions reported in the literature, the yields obtained in this NADES system were higher or comparable.

Considering the complexity of the Maillard reaction, the ability to selectively modulate its intermediates, kinetics, and final products is critically important. The NADES-based ATPS approach provides a structured microenvironment to control these transformations. This opens new opportunities to optimize the synthesis of desirable Maillard-derived compounds while mitigating undesirable byproduct formation, advancing the precision design of flavor, color, and bioactive properties in complex food and biotechnological applications. In this context, the focus of this study was to develop NADES-K_2_HPO_4_ ATPSs and explore their possible applications for accomplishing condensation reactions. The Maillard reaction was assessed as a model to evaluate the potential of ATPSs in facilitating and controlling chemical transformations. Given that the partitioning of reactants in biphasic systems affects the Maillard reaction pathway and product yield, we hypothesized that NADES-K_2_HPO_4_ ATPSs could influence the yield of Amadori compounds.^16,17^ Four types of NADESs, including betaine-glycerol (Bet:Gly), betaine-propylene glycol (Bet:PG), choline chloride-glycerol (ChCl:Gly), and choline chloride-propylene glycol (ChCl:PG), were tested with different salt concentrations. This was followed by characterization of NADES-K_2_HPO_4_ ATPSs density, viscosity, pH, water activity, and thermal behavior. Then, the partitioning of Maillard reactants (glucose, alanine, and phenylalanine) in NADES-K_2_HPO_4_ ATPSs was evaluated. Finally, the potential of NADES-K_2_HPO_4_ ATPSs in accomplishing the Maillard reaction was investigated.

## Materials and Methods

### Materials

Choline chloride (≥99.0%, CAS No. 67–48–1), glycerol (≥99.5%, CAS No. 56–81–5), betaine anhydrous (≥98.0%, CAS No. 107–43–7), dipotassium hydrogen phosphate trihydrate (K_2_HPO_4_·3*H*_2_ *O*, ≥99.0%, CAS No. 16788–57–1), propylene glycol (1,2-propanediol, ≥99.5%, CAS No. 57–55–6), l-alanine (CAS No. 56–41–7), l-phenylalanine (CAS No. 63–91–2), and *o*-phthaldialdehyde (OPA, CAS No. 643–79–8) reagent were purchased from Merck (Darmstadt, Germany). The Amadori compounds *N*-(1-deoxy-d-fructos-1-yl)-l-alanine (Fru-Ala, CAS No. 16124–24–6) and *N*-(1-deoxy-d-fructos-1-yl)-l-phenylalanine (Fru-Phe, CAS No. 31105–03–0) were obtained from Toronto Research Chemicals (Toronto, Canada). Chloride assay kit, glycerol assay kit, and phosphate colorimetric kit were obtained from Merck. All of the other chemicals used in the present study were of analytical grade.

### Preparation of NADESs

NADESs were prepared by combining the components in the precise molar ratios outlined in Table 1, without adding water. The NADES consisted of a hydrogen bond acceptor, either betaine or choline chloride, and a hydrogen bond donor, either glycerol or propylene glycol. Each mixture was heated in a water bath at temperatures ranging from 50 to 80 °C with continuous stirring at 1000 rpm until a clear homogeneous liquid was obtained, and then stored at 25 °C for further use.^3^ The homogeneity of NADES was confirmed by visual inspection with no phase separation or precipitation observed within 24 h of storage at room temperature (25 °C).

**Table 1:** Composition and Preparation Conditions of NADESs

abbreviation	combination	molar ratio	temperature (°C)	time (h)	refs
ChCl:Gly	choline chloride/glycerol	1:2	80	1	^18^
ChCl:PG	choline chloride/propylene glycol	1:3	50	1	^3^
Bet:Gly	betaine/glycerol	1:2	80	1	^19^
Bet:PG	betaine/propylene glycol	1:3	80	1.5	^4^

### Nuclear Magnetic Resonance (NMR)

NMR spectra were recorded on an Avance III NMR spectrometer (Bruker, Billerica, MA) operating at 500 MHz equipped with a 5 mm ^1^H/^13^C TXI probe. Samples were measured neat in 5 mm NMR tubes equipped with a coaxial insert. This insert was filled with deuterated acetone ([CD_3_]_2_CO) with a drop of nonlabeled acetone to allow locking, shimming, and aligning the spectra. Standard proton spectra, COSY, HSQC, and carbon spectra were recorded using standard Bruker pulse programs.

### Preparation of NADES-K_2_HPO_4_ ATPS

After the NADESs were prepared, aqueous two-phase systems were made by combining K_2_HPO_4_ solution with one of the prepared NADESs. A series of K_2_HPO_4_ solutions with concentrations ranging from 62.7 to 7.0 wt % were prepared by dissolving K_2_HPO_4_·3H_2_O in Milli-Q water. The highest concentration, 62.7 wt %, was selected as the starting point based on the work of Marappan and co-workers,^8^ who reported binodal curves for various NADES–K_2_HPO_4_ ATPS. The K_2_HPO_4_ solutions were then mixed with NADESs in a volume ratio of 1:1 to obtain a total volume of 4 mL. The mixtures were shaken thoroughly and then allowed to phase separate under static conditions at 25 °C for 1 h. The selected concentrations of K_2_HPO_4_ for each NADES were as follows: 54.7 wt % for Bet:Gly, 62.7 wt % for Bet:PG, and 40.8 wt % for ChCl:PG. The volume ratio of ATPS after phase separation was calculated by

1 $$v\mathrm{olume ratio}=V_{\mathrm{top}}/V_{\mathrm{bottom}}$$

where *V*_top_ and *V*_bottom_ represent the volume of the top phase and bottom phase, respectively.

### Water Content Ratio and Water Phase Separation Efficiency

Water content ratio was calculated based on the water signal measured in ^1^H NMR with acetone, present in the coaxial insert of the NMR tube and used as an internal standard. The ratio represents the amount of water in the top phase relative to the amount in the bottom phase. Water phase separation efficiency was calculated based on the volume ratio and water content ratio, reflecting the percentage of water in the top phase.

These two indices were calculated as follows

2 $$w\mathrm{ater content ratio}=\frac{S_{water\;in\;top}}{S_{acetone\;in\;top}}/\frac{S_{water\;in\;bottom}}{S_{acetone\;in\;bottom}}$$

3 $$w\mathrm{ater phase separation efficiency}=\frac{S_{\mathrm{water}\;\mathrm{in}\;\mathrm{top}}\times V_{\mathrm{top}}}{S_{\mathrm{acetone}\;\mathrm{in}\;\mathrm{top}}}/\frac{S_{\mathrm{water}\;\mathrm{in}\;\mathrm{top}}\times V_{\mathrm{top}}}{S_{\mathrm{acetone}\;\mathrm{in}\;\mathrm{top}}}+\frac{S_{\mathrm{water}\;\mathrm{in}\;\mathrm{bottom}}}{S_{\mathrm{acetone}\;\mathrm{in}\;\mathrm{bottom}}}\times V_{\mathrm{bottom}}$$

where *S*_water in top_ and *S*_acetone in top_ represent the intensities of the water and acetone signals, respectively, in the top phase, and *S*_water in bottom_ and *S*_acetone in bottom_ denote the corresponding signal intensities in the bottom phase. Similarly, *V*_top_ and *V*_bottom_ represent the volumes of the top and bottom phases, respectively.

### The Partitioning of Components in NADES-K_2_HPO_4_ ATPS

After phase separation, the concentrations of choline chloride, glycerol, and K_2_HPO_4_ in the top and bottom phases were measured using a chloride assay kit, glycerol assay kit, and phosphate colorimetric kit, respectively.

The quantification of betaine was performed using a colorimetric ultraviolet–visible (UV–vis) method based on its reaction with Reinecke’s salt under strongly acidic conditions, following a previously reported method with slight modifications.^20^ The reaction produces a colored complex that exhibits a characteristic absorbance at 525 nm. The Reinecke’s salt solution was freshly prepared by dissolving 1.50 g of ammonium reineckate in 100 mL of distilled water and adjusting the pH to 1.0 using hydrochloric acid. The solution was stirred at room temperature for 45 min and then filtered before use. The diethyl ether wash solution was prepared by diluting reagent-grade, alcohol-free diethyl ether with 1 mL of distilled water per 140 mL of ether. For analysis, 1 mL of each betaine-containing sample (diluted and adjusted to pH 1.0 with 6 M HCl) was mixed with 1 mL of the freshly prepared Reinecke’s salt solution in a 2 mL centrifuge tube. The mixture was incubated at 4 °C for 1.5 h to allow for complete precipitation. It was then centrifuged at 9391*g* for 15 min, and the supernatant was discarded. The precipitate was washed with 1 mL of diethyl ether wash solution to facilitate crystal transfer and effectively displace residual mother liquor without dissolving the target complex, followed by a second centrifugation at 9391*g* for 10 min. The residual ether was evaporated under ambient conditions in a fume hood. The resulting dry residue was redissolved in 1 mL of 70% acetone, and the absorbance was measured at 525 nm. A standard calibration curve was prepared by using betaine hydrochloride solutions ranging from 0 to 8.538 mM.

The quantification of propylene glycol was carried out using a gas chromatography system equipped with a flame ionization detector (GC-FID, GC-2014, Shimadzu, Kyoto, Japan). The method was adapted from a previously published protocol originally developed for the analysis of short-chain and branched-chain fatty acids, with modifications to suit the detection of propylene glycol.^21^ Samples were diluted with methanol and then filtered through a regenerated cellulose filter (15 mm Ø, 0.2 μm, Phenomenex, Torrance, CA). Nitrogen was used as a carrier gas. Samples were injected in the splitless mode. The injector and detector temperatures were set at 250 °C. The initial column temperature was set to 100 °C and held for 0.035 min, after which it was ramped up to 172 °C at a rate of 10.8 °C/min. The temperature then increased at a rate of 50 °C/min to 200 °C, and it was maintained at 200 °C for 1 min. A standard calibration curve for propylene glycol was prepared, covering a concentration range of 0–1.315 mM. The limit of detection was determined to be 54 μM.

The partition coefficient (*K*) of components was calculated by

4 $$K=C_{\mathrm{top}}/C_{\mathrm{bottom}}$$

where *C*_top_ and *C*_bottom_ represent the concentrations of a component at the top and bottom phases, respectively.

The partition extent (*E*_top_) of a given NADES component at the top phase was calculated as

5 $$E_{\mathrm{top}}=C_{\mathrm{top}}\times V_{\mathrm{top}}/\left(C_{\mathrm{top}}\times V_{\mathrm{top}}+C_{\mathrm{bottom}}\times V_{\mathrm{bottom}}\right)$$

where *C* denotes the component concentration, *V* represents phase volume, and the subscripts correspond to the top and bottom phases. *C* and *V* in eqs 4 and 5 are given in milligrams of mass and mL, respectively.

### Physicochemical Properties

The physicochemical properties of each phase of NADES-K_2_HPO_4_ ATPSs were investigated using NADESs (ChCl:PG; Bet:Gly; Bet:PG) and K_2_HPO_4_ solutions as references. The pH values were determined at 25 °C using a 1000L pH meter (VWR, Darmstadt, Germany). The density of the top and bottom phases was measured at 25 °C through the volumetric method and reported in g/mL, and their water activity was measured by a LabMaster-aw instrument (Novasina, Horsham, U.K.) at 25 °C. The viscosity of phases was measured at 25 °C using an MCR 302e rheometer (Anton Paar, Austria) equipped with a concentric cylinder geometry, and reported in mPa·s.^22^ Differential scanning calorimetry (DSC) was performed on the temperature range from −80 to 20 °C at a rate of 10 °C/min, under nitrogen atmosphere.^3^ Pure substances, including propylene glycol, betaine, choline chloride, glycerol, K_2_HPO_4_, and water, were also analyzed by DSC to determine their thermal properties.

### The Partitioning of Amino Acids in NADES-K_2_HPO_4_ ATPSs

The amino acids alanine and phenylalanine were individually introduced into NADES-K_2_HPO_4_ ATPSs at a final concentration of 0.01 mol/L, followed by stirring the samples until complete dissolution. The samples were subsequently allowed to phase separate (no stirring) at 25 °C for 4 h. After phase separation, the amino acids were quantified by the OPA at both phases.^23^

### Microscopic Imaging of the Emulsion Droplets

The Bet:PG-K_2_HPO_4_ ATPS was supplemented with 0.01 mg/mL fluorescein-5-isothiocyanate (FITC). FITC is hydrophobic (log *P* = 5.25) and can partition into the Bet:PG-rich phase.^24^ The mixture was stirred at 1000 rpm to ensure complete dissolution. The ATPS microstructure was then imaged on glass slides by using a fluorescence microscope (Leica DMi8, Leica Microsystems, Heerbrugg, Germany). Imaging was performed to identify which phase formed the dispersed phase of the ATPS.

### Running the MR in ATPSs and Single-Phase Solutions

The Bet:PG-K_2_HPO_4_ ATPSs were supplemented with the Maillard reactants (glucose and either alanine or phenylalanine) at a final concentration of 40 mM. Subsequently, the mixtures were stirred for 3 h at 25 °C to ensure complete dissolution.

The reactant-supplemented ATPS samples (4 mL each) were transferred into 10 mL gas chromatography vials, which were then sealed. The samples were incubated at 37 °C for 0, 3, and 5 days while being stirred at 1000 rpm. Afterward, the samples were cooled and stored at 25 °C for 4 h to allow complete phase separation. Next, the top and bottom phases were collected, ultrafiltered, and analyzed. The phases were designated as Top-priorPS (i.e., the top phase from the ATPS underwent reaction prior to phase separation) and Bottom-priorPS (i.e., the bottom phase from the ATPS underwent reaction prior to phase separation). Alternatively, the whole ATPS was analyzed before the phase separation. For comparison purposes, the reactants were introduced into the top and bottom phases after ATPS phase separation and were designated as Top-postPS (i.e., top phase from the ATPS underwent reaction after phase separation) and Bottom-postPS (i.e., bottom phase from the ATPS underwent reaction after phase separation), which served as control groups (Figure 1). Additionally, solutions of reactants (glucose, phenylalanine, and alanine) in water were prepared as controls. All samples were prepared in two independent preparations, with each condition prepared in duplicate to ensure reproducibility. Portions of the samples were stored at −20 °C for later analysis, whereas others were analyzed immediately.

**Figure 1:**
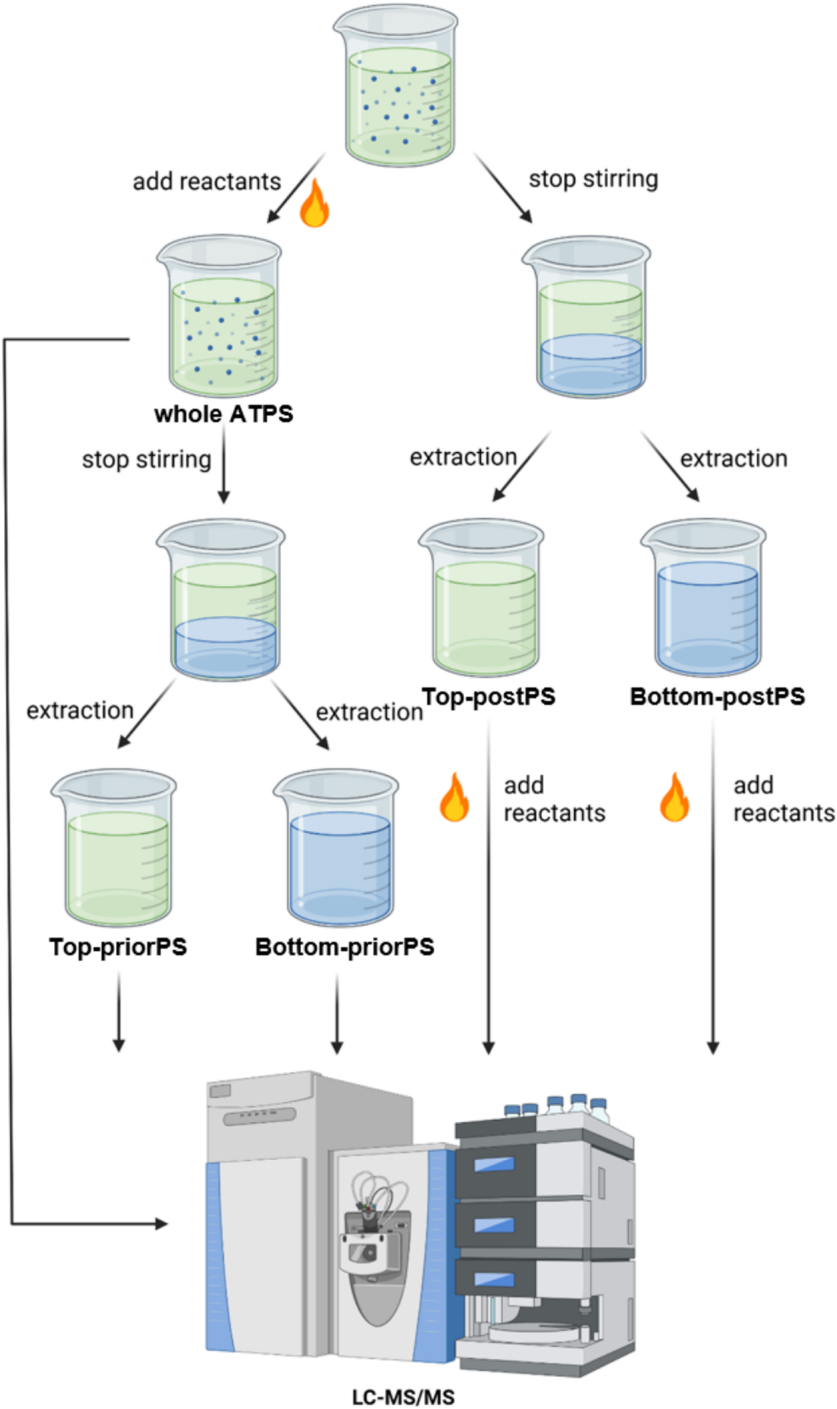
Schematic diagram of sample preparation and analysis for the Maillard reaction. Control solutions were prepared by dissolving the reactants (glucose, alanine, and phenylalanine, alternatively) in water (not shown in the diagram). This diagram was created with BioRender.

### Liquid Chromatography–High-Resolution Tandem Mass Spectrometry

ATPSs, their respective top and bottom phases, and control solutions were diluted in water, centrifuged (5 min, 18,000*g*, 4 °C), and finally diluted 100 times in 80% v/v acetonitrile. Liquid chromatography–high-resolution tandem mass spectrometry (LC-MS/MS) data were acquired using an Exploris 120 quadrupole Orbitrap mass spectrometer interfaced to a Vanquish liquid chromatographic system (Thermo Fisher Scientific, Bremen, Germany). Target compounds (alanine, phenylalanine, glucose, *N*-[1-deoxy-d-fructos-1-yl]-l-alanine, *N*-[1-deoxy-d-fructos-1-yl]-l-phenylalanine) were separated at 35 °C through a zwitterionic sulfobetaine column (Atlantis Premier BEH, Z-HILIC, 100 mm × 2.1 mm, 1.7 μm, Waters, Milford MA) with the following gradient of solvent B (minutes/%B): (0/2), (2/2), (13/50), (14/35), (15/35). Mobile phases consisted of acetonitrile/water, 90:10 v/v with 5 mM ammonium formate and 0.1% formic acid (solvent A) and water/acetonitrile, 95:5 v/v with 5 mM ammonium formate and 0.1% formic acid (solvent B). The flow rate was 0.25 mL/min. H-ESI interface parameters were as follows: static spray voltage 3.3 and −3.0 kV for positive and negative ion mode, respectively; ion transfer tube and vaporizer temperatures were set at 325 and 350 °C; sheath gas flow and auxiliary gas flow were 50 and 10 arbitrary units, respectively. Compounds were identified and quantified in targeted MS/MS mode screening the precursor ions according to a mass list generated in Trace Finder (v. 5.1, Thermo Fisher Scientific, Waltham, MA) and including (chemical formula, ionization, mass to charge ratio, linearity range): alanine (C_3_H_7_NO_2_, [M + H]^+^: *m*/*z* 90.0550, 0.56–112.00 μM), phenylalanine (C_9_H_11_NO_2_, [M + H]^+^: *m*/*z* 166.0863, 0.30–60.00 μM), *N*-(1-deoxy-d-fructos-1-yl)-l-alanine (C_9_H_17_NO_7_, [M + H]^+^: *m*/*z* 252.1078, 0.20–40.00 μM), *N*-(1-deoxy-d-fructos-1-yl)-l-phenylalanine (C_15_H_21_NO_7_, [M + H]^+^: *m*/*z* 328.1391, 0.15–30.60 μM), and glucose (C_6_H_12_O_6_, [M – H]^−^ *m*/*z* 179.0561, 0.27–55.55 μM). For tandem MS experiments ion scan, normalized collision energy was set to 35%, Orbitrap resolution at 30,000 (fwhm at *m*/*z* 200), and the quadrupole resolution was set at 1.2. Calibration curves were built by using pure reference standards, while analytical performances were monitored by using accurate masses and an error within ±2 ppm for precursor and fragment ions. The limit of detection was measured by using reference standards in 80% acetonitrile (v/v), and it was 0.01 μM for alanine and phenylalanine, 0.05 μM for glucose, and 0.02 μM for the two Amadori compounds.

### Statistical Analysis

Samples were prepared independently on two separate occasions, with two duplicates prepared each time. For each sample, measurements were performed in duplicate. Data are presented as the mean ± standard deviation (SD). Where appropriate, error bars in the figures represent the SD of the replicates. The results were analyzed using SPSS Statistics 28.0 (IBM software, New York, NY). Data were tested for the normality and homogeneity of variances. Then, one-way ANOVA was used to evaluate statistical differences among the sample groups. Tukey’s test was first applied to compare differences among samples for the pH, viscosity, water activity, and density. In addition, comparisons among all sample groups were conducted at each incubation time point to evaluate intersample differences at the same time in the concentration of glucose, alanine, phenylalanine, and the two Amadori compounds. Comparisons across different incubation time points were performed within each sample group to assess temporal changes. The significance level was set at *P* < 0.05.

## Results and Discussion

### NADES Characterization

Figure 2 displays the ^1^H NMR spectra of ChCl:Gly, ChCl:PG, Bet:Gly, and Bet:PG. The ^1^H NMR spectra of pure compounds (choline chloride, betaine, glycerol, and propylene glycol), along with the ^13^C NMR and ^1^H–^13^C HSQC spectra of both pure compounds and NADES, and the ^1^H–^1^H COSY spectra of NADES are presented in Figures S1–S14 (Supporting Information). In the spectrum of ChCl:Gly (Figure 2a), the signals corresponding to the hydrogens on the methylene group bound to nitrogen (indicated with b) and the hydrogens attached to the terminal carbons of glycerol (indicated with f) overlap, consistent with the observations of Delso, Lafuente, Muñoz-Embid, and Artal.^25^ The calculated molar ratio of ChCl to Gly was 1:2.09. In the spectrum of ChCl:PG (Figure 2b), the peaks were resolved and identified. Integration of the ^1^H NMR spectrum indicated that the molar ratio of ChCl to PG was 1:2.91. As shown in Figure 2c, the peaks for the hydrogens on the terminal carbon atoms (indicated with d) and the hydrogen on the central carbon atom (indicated with e) of Gly overlapped. In this case, the integration of the ^1^H NMR spectrum yielded a Bet/Gly molar ratio of 1:2.37. For Bet:PG, the peaks are well resolved and identified (Figure 2d). The calculated molar ratio of Bet to PG was 1:2.94. The calculated molar ratios of all four NADESs matched their initial values, as presented in Table 1, confirming that no reactions occurred between the components.

**Figure 2:**
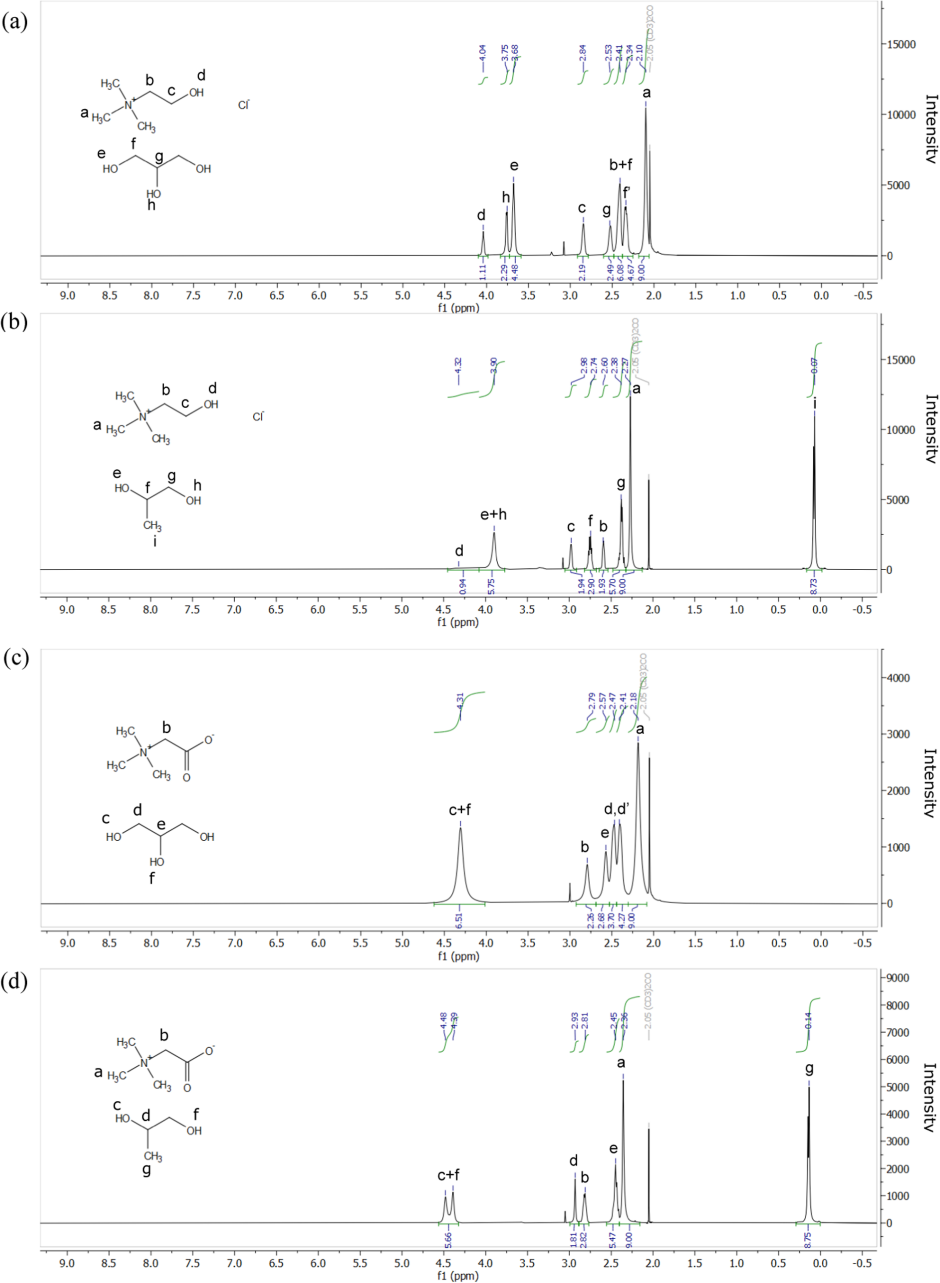
^1^H NMR spectra of (a) ChCl: Gly, (b) ChCl: PG, (c) Bet:Gly, and (d) Bet:PG.

The NADESs were further characterized using DSC to identify thermal transitions, and the results were compared to those of the raw materials.^26^ The analyses were conducted over a temperature range of −80 to 20 °C. Prior to examining the mixtures, pure compounds were analyzed, and their respective thermograms are presented in Figure 3. For ChCl, Bet, and PG, no thermal events were detected within this temperature range. The DSC thermogram of Gly, however, revealed a glass transition at approximately −80 °C, which aligns well with previous findings.^27^

**Figure 3:**
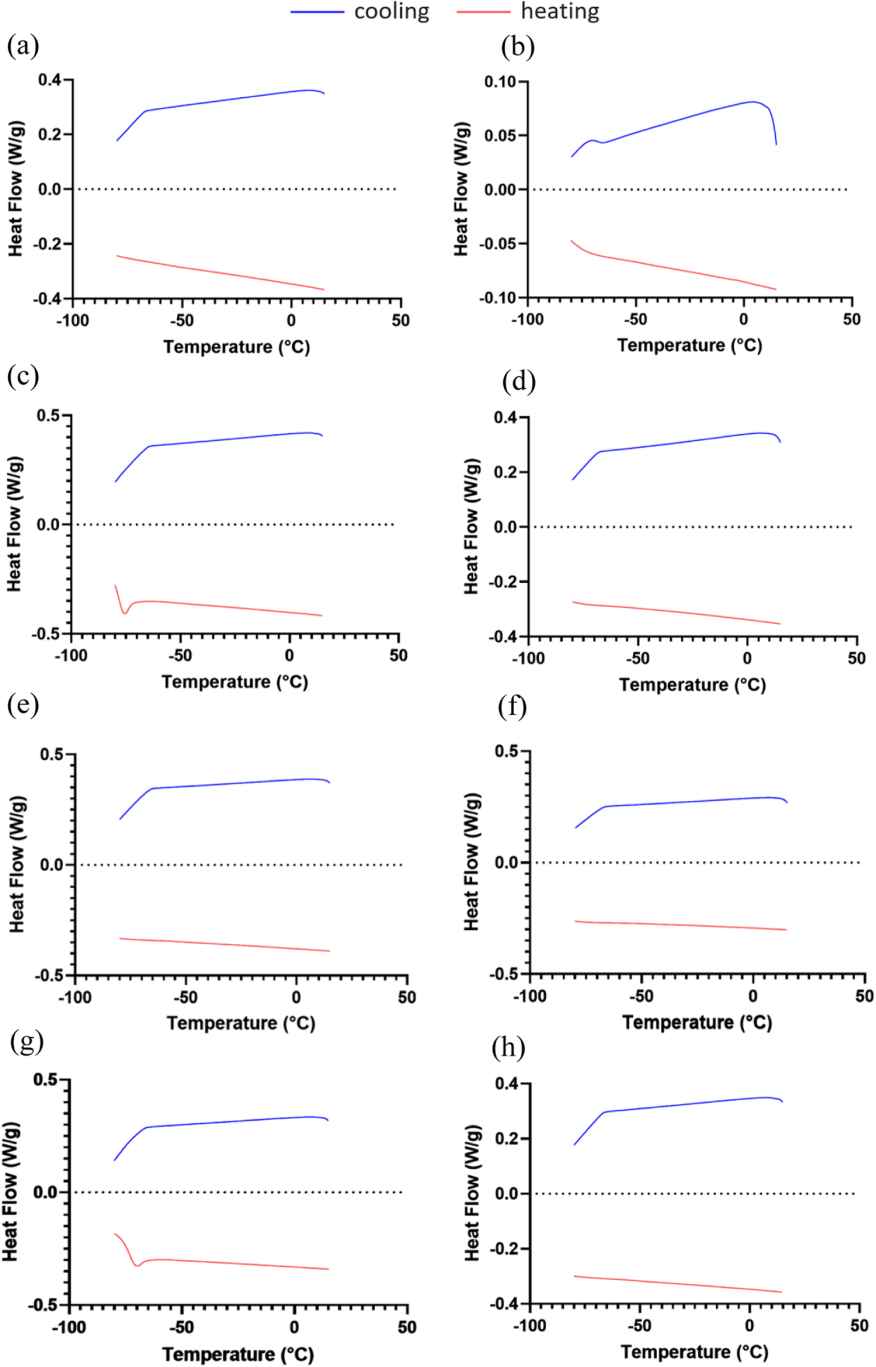
Heat flow thermograms obtained at 10 °C/min for pure compounds and NADESs: (a) ChCl, (b) Bet, (c) Gly, (d) PG, and (e) ChCl:Gly, (f) ChCl:PG, (g) Bet:Gly, and (h) Bet:PG.

The DSC thermogram of Bet:Gly exhibited only a glass transition at approximately −74.2 °C, as shown in Figure 3g. Conversely, no glass transition was observed for ChCl:Gly, ChCl:PG, and Bet:PG during heating from −80 to 20 °C (Figure 3e,f,h). It is possible that the glass transitions for these samples occur at temperatures below the detection limit of our equipment (−80 °C).^28^ Similarly, Mero, Koutsoumpos, Giannios, Stavrakas, Moutzouris, Mezzetta, and Guazzelli observed no glass transitions when analyzing ChCl:Gly with molar ratios of 1:2, 1:3, and 1:4 at a temperature range of −90 to 100 °C.^29^ Some NADESs have been reported to exhibit glass transition points below −100 °C. Examples include ChCl-ethylene glycol (1:2), ChCl-Gly-water (1:2:1), and ChCl-PG-water (1:1:1).^3,30^ Our results suggest that the four NADESs examined are supramolecular complexes that remain stable in the liquid state across a broad temperature range, making them suitable for use as solvents under diverse conditions.

### Formation of NADES-K_2_HPO_4_ ATPS

NADESs were mixed with different concentrations of K_2_HPO_4_ solutions in a volume ratio of 1:1. As presented in Table 2, liquid–liquid (L-L) two-phase systems were observed in the mixtures of Bet:Gly-K_2_HPO_4_, Bet:PG-K_2_HPO_4_, and ChCl:PG-K_2_HPO_4_. The lowest K_2_HPO_4_ concentrations allowing phase separation in Bet:Gly-K_2_HPO_4_, Bet:PG-K_2_HPO_4_, and ChCl:PG-K_2_HPO_4_ were 54.7, 17.9, and 25.1 wt %, respectively. Therefore, the ability of NADESs to form an ATPS together with K_2_HPO_4_ follows the trend: Bet:PG > ChCl:PG > Bet:Gly. It has been reported that hydrophilicity of NADES is one of the key factors influencing phase formation.^31^ The logarithm of octanol–water partition coefficients (log *P*) of choline chloride, betaine, glycerol, and propylene glycol are −4.66, −4.49, −1.76, and −0.92, respectively.^32–34^ The Bet:PG combination exhibits the highest hydrophobicity among the four NADESs. Both Bet:PG and ChCl:PG are hydrophobic NADESs synthesized from poorly water-soluble components. In the formulation of hydrophobic NADESs, HBAs can be either ionic (e.g., choline chloride, betaine) or nonionic (e.g., monoterpenes), while common HBDs include phenols, alcohols, and glycols.^35^ The difference in hydrophobicity between the two phases of ATPSs was a major driving force behind the phase separation; the use of the Bet:PG combination led to the most significant dissimilarity, resulting in the smallest amount of K_2_HPO_4_ needed to induce phase separation.^8^ As the concentration of K_2_HPO_4_ increased, three distinct systems were observed for ChCl:PG-K_2_HPO_4_ and Bet:Gly-K_2_HPO_4_ mixtures: a single-phase liquid, a liquid–liquid two-phase system, and a single-phase liquid together with sediment formation (Table 2). The sediment formation was attributed to the limited solubility of K_2_HPO_4_.^2^

**Table 2:** Sample Final State after Mixing Dipotassium Phosphate Solutions and NADESs at a Volume Ratio of 1:1^a^

K_2_HPO_4_ concentration (wt %)	ChCl:Gly	ChCl:PG	Bet:Gly	Bet:PG
62.7	-	-	-	L/L
59.3	-	-	-	L/L
58.0	-	-	S-L	L/L
56.3	-	-	S-L	L/L
54.7	S-L	-	L/L	L/L
52.9	S-L	-	L	L/L
51.1	S-L	-	L	L/L
49.2	S-L	-	L	L/L
47.2	S-L	S-L	L	L/L
45.2	S-L	S-L	L	L/L
43.0	S-L	S-L	L	L/L
40.8	S-L	L/L	L	L/L
38.5	S-L	L/L	L	L/L
36.0	L	L/L	-	L/L
33.5	L	L/L	-	L/L
30.8	L	L/L	-	L/L
28.1	-	L/L	-	L/L
25.1	-	L/L	-	L/L
22.8	-	L	-	L/L
20.9	-	L	-	L/L
17.9	-	L	-	L/L
15.7	-	L	-	L
12.5	-	L	-	L
10.5	-	L	-	L
9.0	-	L	-	L
7.0	-	L	-	L

a Notes: (a) ‘S-L’ denotes sediment formation in single phase. (b) ‘L/L’ represents liquid/liquid two phases. (c) ‘L’ denotes single liquid phase. (d) ‘-’ defines the experiment for the K_2_HPO_4_ concentration was not included.

Remarkably, it was observed that ATPS was not formed in any of the ChCl:Gly-K_2_HPO_4_ mixtures. ChCl:Gly-K_2_HPO_4_ formed sediment at the K_2_HPO_4_ concentration range between 54.7 and 38.5 wt % and then transitioned directly to single-phase liquid when K_2_HPO_4_ concentration reached 36.0%. These findings contradict the results of Marappan, Kamaruddin, and Gonawan, who reported the formation of ATPS with K_2_HPO_4_ concentrations ranging from 10 to 50 wt % and ChCl:Gly concentrations ranging from 80 to 30 wt %.^8^ A possible explanation for this discrepancy might be the different sample preparation methods. They used a titration technique in which a K_2_HPO_4_ solution (1 g/mL) was added dropwise to ChCl:Gly NADES until a two-phase system was observed. In contrast, in our study, samples were prepared by mixing ChCl:Gly NADES and a K_2_HPO_4_ solution at fixed concentrations and a 1:1 volume ratio. The controlled conditions in the titration method may facilitate phase separation, whereas the fixed concentration method may not.

Based on the data presented in Table 2, we selected specific K_2_HPO_4_ concentrations for each combination: 40.8 wt % for ChCl:PG, 54.7 wt % for Bet:Gly, and 62.7 wt % for Bet:PG. These concentrations were chosen to minimize water content within the system, as excessive dilution of NADES can disrupt hydrogen-bonding networks and compromise the supramolecular structure.^12^ Dai, Witkamp, Verpoorte, and Choi demonstrated that in ChCl/PG/water (1:1:1), the supramolecular complex structure of the NADES remained intact when water content was below 50% (v/v). However, further dilution led to the dissociation of NADES into free forms of its individual components in water.

### Phase Composition of the ATPSs

In the ChCl:PG-K_2_HPO_4_ ATPS, the partition coefficient (K) of ChCl was approximately 6, while PG exhibited a significantly higher K of 26.27 (Table 3). In this system, about 22% of K_2_HPO_4_ remained in the top phase. In the Bet:PG-K_2_HPO_4_ ATPS, betaine concentration in the top phase was approximately six times higher than that in the bottom phase, and the K of PG reached an impressive value of 1171.60. This reflects the system’s high selectivity and efficient separation capability, with nearly complete PG migration (∼99.9%) into the top phase and only ∼2% of K_2_HPO_4_ retained there. In the Bet:Gly-K_2_HPO_4_ ATPS, the top phase was rich in betaine, while the bottom phase contained a higher concentration of K_2_HPO_4_ (Table 3). Unlike the other two NADES-K_2_HPO_4_ ATPSs, the HBD in the Bet:Gly-K_2_HPO_4_ ATPS, namely, glycerol, the corresponding *K* value of 1.44 suggests a near-equilibrium distribution of glycerol, with approximately 59% of glycerol partitioning into the top phase. The difference in the preferential partitioning of the HBDs, including PG and glycerol, between the two phases can be attributed to their different hydrophobicity. The LogP values of PG and glycerol are −0.92 and −1.76, respectively.^32^ In summary, the top and bottom phases of Bet:PG-K_2_HPO_4_ ATPS and ChCl:PG-K_2_HPO_4_ ATPS were NADES-rich and K_2_HPO_4_-rich, respectively, while the Bet:Gly showed a more even distribution of the components over the two phases.

**Table 3:** Concentration of Components in Each Phase and Their Partition Coefficient (K) and Partition Extent at the Top Phase (*E*_top_) in Three NADES-K_2_HPO_4_ ATPSs

ATPSs	components	concentration in the top phase (mM)	concentration in the bottom phase (mM)	*K*	*E*_top_ (%)
ChCl:PG-K_2_HPO_4_	choline chloride	1913.39 ± 49.23	319.69 ± 9.04	5.99 ± 0.02	94.73 ± 0.02
	propylene glycol	5737.14 ± 21.49	218.68 ± 8.04	26.27 ± 1.06	98.75 ± 0.05
	K_2_HPO_4_	422.17 ± 2.66	4629.47 ± 234.24	0.09 ± 0.01	21.52 ± 1.36
Bet:Gly-K_2_HPO_4_	betaine	2770.91 ± 29.24	607.95 ± 20.47	4.20 ± 0.12	70.87 ± 0.37
	glycerol	4862.77 ± 993.63	3425.77 ± 117.44	1.42 ± 0.07	58.67 ± 1.19
	K_2_HPO_4_	1419.29 ± 94.86	4007.67 ± 234.90	0.36 ± 0.03	31.97 ± 1.93
Bet:PG-K_2_HPO_4_	betaine	3393.33 ± 271.97	560.39 ± 14.62	6.06 ± 0.46	88.89 ± 0.74
	propylene glycol	9482.02 ± 470.48	8.11 ± 0.65	1171.60 ± 36.39	99.94 ± 0.00
	K_2_HPO_4_	183.14 ± 17.04	7448.89 ± 283.09	0.03 ± 0.00	2.41 ± 0.28

### Water Distribution in the ATPSs

The total water content of the ATPSs and the volume ratio between the top and bottom phases of the ATPSs are presented in Table 4. The larger proportion of the top phase in ChCl:PG-K_2_HPO_4_ ATPS is most likely due to the lower concentration of K_2_HPO_4_ (23.0 wt %) used in making this ATPS, which allowed ChCl:PG to retain more water in the top phase.

**Table 4:** Volume Ratio of Selected NADES-K_2_HPO_4_ ATPS after Phase Separation and Their Overall Composition

NADES-K_2_HPO_4_ ATPS			overall composition (wt %)		
component 1	component 2	volume ratio (top phase/bottom phase)	NADES	K_2_HPO_4_	water
ChCl:PG	40.8 wt % K_2_HPO_4_	1.5:0.5	43.6	23.0	33.4
Bet:Gly	54.7 wt % K_2_HPO_4_	0.98:1.02	43.8	30.7	25.5
Bet:PG	62.7 wt % K_2_HPO_4_	1.14:0.86	39.2	38.1	22.7

The water content ratio between the top and bottom phases was approximated using water signal in the ^1^H NMR spectra (Figure S15), and the results are given in Table 5. Among the evaluated systems, the Bet:Gly-K_2_HPO_4_ ATPS exhibited a water content ratio greater than 1, indicating that most of the water in the K_2_HPO_4_ solution transferred into the top phase. It is noteworthy that the top phase of this system was only HBA-rich, and glycerol (as HBD) partitioned almost equally between the two phases (Table 3). In contrast, the water content ratio of the Bet:PG-K_2_HPO_4_ and ChCl:PG-K_2_HPO_4_ ATPSs was approximately 0.5 (Table 5), indicating that the NADES-rich top phases that were rich in both HBA and HBD could not efficiently transfer water from the bottom phase. The inability of the top phases rich in both HBA and HBD, in comparison to that only rich in HBA, to transfer water from the bottom phase was attributed to the hydrophobicity of the HBD component.

**Table 5:** Water Content Ratio and Water Phase Separation Efficiency in Three NADES-K_2_HPO_4_ ATPSs

ATPSs	water content ratio^a^	water phase separation efficiency^b^
ChCl:PG-K_2_HPO_4_	0.515 ± 0.011	60.709 ± 0.515
Bet:Gly-K_2_HPO_4_	1.137 ± 0.062	52.176 ± 1.368
Bet:PG-K_2_HPO_4_	0.473 ± 0.003	38.525 ± 0.128

a Water content ratio represents the ratio of water content in the top phase relative to that in the bottom phase. This index was calculated according to the water signal determined in ^1^H NMR using acetone as an internal standard.

b Water phase separation efficiency refers to the percentage of water in the top phase. This index was calculated according to the volume ratio and water content ratio.

### Physical Properties

The physical properties of NADESs, K_2_HPO_4_solutions, and NADES-K_2_HPO_4_ ATPSs, including density, pH, water activity, and viscosity, were investigated and are reported in Table 6. Among the NADESs, Bet:Gly exhibited the highest density (∼1.25 g/mL), followed by Bet:PG (∼1.10 g/mL) and ChCl:PG (∼1.09 g/mL). The densities of the top phase of both ChCl:PG-K_2_HPO_4_ and Bet:PG-K_2_HPO_4_ ATPS were significantly lower than those of their corresponding bottom phases. This remarkable disparity is attributed to the high concentration of K_2_HPO_4_ in the bottom phase, which was ∼4629 mM for ChCl:PG-K_2_HPO_4_ and ∼7448 mM for Bet:PG-K_2_HPO_4_ (Table 3).

**Table 6:** Physical Properties of NADES-K_2_HPO_4_ ATPS Determined at 25 °C with NADESs and K_2_HPO_4_ Solutions as References^a^

sample	density (g/mL)	pH	water activity	viscosity (mPa·s)
ChCl:PG	1.096 ± 0.008^a^	7.564 ± 0.062^a^	0.099 ± 0.007^b^	65.709 ± 0.207^e^
Bet:Gly	1.256 ± 0.001^b^	8.610 ± 0.113^b^	0.054 ± 0.002^a^	2300.013 ± 43.359^j^
Bet:PG	1.106 ± 0.001^a^	9.550 ± 0.002^c^	0.084 ± 0.005^b^	168.320 ± 1.694^f^
40.8 wt % K_2_HPO_4_ solution	1.420 ± 0.007^c^	9.633 ± 0.007^c^	0.801 ± 0.016^i^	4.954 ± 0.197^a^
54.7 wt % K_2_HPO_4_ solution	1.611 ± 0.026^d^	10.113 ± 0.003^d^	0.646 ± 0.007^h^	15.497 ± 0.454^b^
62.7 wt % K_2_HPO_4_ solution	1.713 ± 0.001^e^	10.455 ± 0.007^f^	0.496 ± 0.002^f^	47.357 ± 0.351^d^
ChCl:PG-K_2_HPO_4_ ATPS top phase	1.103 ± 0.028^a^	10.261 ± 0.001^de^	0.567 ± 0.013^g^	14.490 ± 0.294^b^
ChCl:PG-K_2_HPO_4_ ATPS bottom phase	1.655 ± 0.008^de^	10.232 ± 0.004^de^	0.576 ± 0.013^g^	27.154 ± 0.390^c^
Bet:Gly-K_2_HPO_4_ ATPS top phase	1.402 ± 0.025^c^	10.405 ± 0.005^ef^	0.405 ± 0.002^de^	425.328 ± 4.188^i^
Bet:Gly-K_2_HPO_4_ ATPS bottom phase	1.479 ± 0.040^c^	10.303 ± 0.035^ef^	0.437 ± 0.008^e^	397.052 ± 4.925^h^
Bet:PG-K_2_HPO_4_ ATPS top phase	1.116 ± 0.005^a^	11.654 ± 0.044^h^	0.373 ± 0.001^cd^	51.712 ± 0.330^d^
Bet:PG-K_2_HPO_4_ ATPS bottom phase	1.878 ± 0.032^f^	11.335 ± 0.012^g^	0.368 ± 0.002^c^	219.820 ± 0.980^g^

a Different letters within a column indicate significant differences between samples (*P* < 0.05).

All three NADESs had alkaline pH values ranging from ∼7.56 to ∼9.55. The pH of NADESs is primarily influenced by the chemical structure of HBDs.^36^ The pH values of K_2_HPO_4_solutions ranged from ∼9.63 to ∼10.46. In accordance with the pH values of both NADES and K_2_HPO_4_ solutions, the pHs of all three NADES-K_2_HPO_4_ ATPSs were alkaline, ranging from ∼10.23 to ∼11.65 (Table 6). The pH values were similar between the top and bottom phases of all ATPSs. These results demonstrate that NADES-K_2_HPO_4_ ATPS systems can establish an alkaline environment, resulting in a valuable tool for optimizing condensation reactions involving amino groups and enhancing the stability of compounds in specific solvents.

As shown in Table 6, the water activity (*a*_w_) value of the three NADESs ranged from ∼0.054 to ∼0.099. Given that no water was added during sample preparation, the *a*_w_ value of 0.099 was likely due to the hygroscopic nature of the neat compounds, particularly choline chloride and betaine that readily absorb moisture from the environment.^37^ All three K_2_HPO_4_ solutions exhibited water activities between = ∼0.496 and = ∼0.801. The *a*_w_ values of the top and bottom phases of all three ATPSs were similar (Table 6). The comparable *a*_w_ values measured despite the observation that the bottom phase of the ChCl:PG-K_2_HPO_4_ and Bet:PG-K_2_HPO_4_ ATPSs had twice as much water content compared to their respective top phases (as approximated from ^1^H NMR spectra) (Table 5) suggests that K_2_HPO_4_ remarkably interacted with water molecules depending on its high hydrophilicity, diminishing the availability of water for vaporization.

Viscosity is a critical variable to consider when working with NADES, as these systems are often characterized by high viscosity values.^38^ Among the NADESs analyzed, Bet:Gly exhibited the highest viscosity with a mean value of 2300 mPa·s, with Bet:PG (168 mPa·s) and ChCl:PG (65 mPa·s) following in decreasing order (Table 6). The high viscosity of NADESs containing glycerol can be a consequence of the inherently high viscosity of glycerol. Additionally, the strong molecular interactions between the HBA and HBD in a deep eutectic solvent restrict molecular mobility, further contributing to its viscosity.^39^ Upon incorporation into ATPS, the viscosity of Bet:Gly decreased significantly to mean values of 425 mPa·s in the top phase and 397 mPa·s in the bottom phase. However, even after this reduction, the viscosity remained notably higher compared to that of the ChCl:PG-K_2_HPO_4_ ATPS and Bet:PG-K_2_HPO_4_ ATPS, which may limit its application. The top phase of each ATPS exhibited a lower viscosity compared to the corresponding NADES. This observation was primarily attributed to the plasticizing effect of water molecules.

### DSC Analysis

As shown in Figure 4, the heat flow thermograms of the top phase of each NADES-K_2_HPO_4_ ATPSs were compared to those of the corresponding NADES. Meanwhile, the heat flow thermograms of the bottom phase (i.e., K_2_HPO_4_-rich) were compared to those of the respective K_2_HPO_4_ solution. No thermal events were observed in ChCl:PG, the top phase of ChCl:PG-K_2_HPO_4_ ATPS, Bet:PG, and the top phase of the Bet:PG-K_2_HPO_4_ ATPS during cooling and heating (Figure 4a,e), indicating that the interaction network of ChCl:PG and Bet:PG may be preserved in the top phase of the corresponding ATPS.

**Figure 4:**
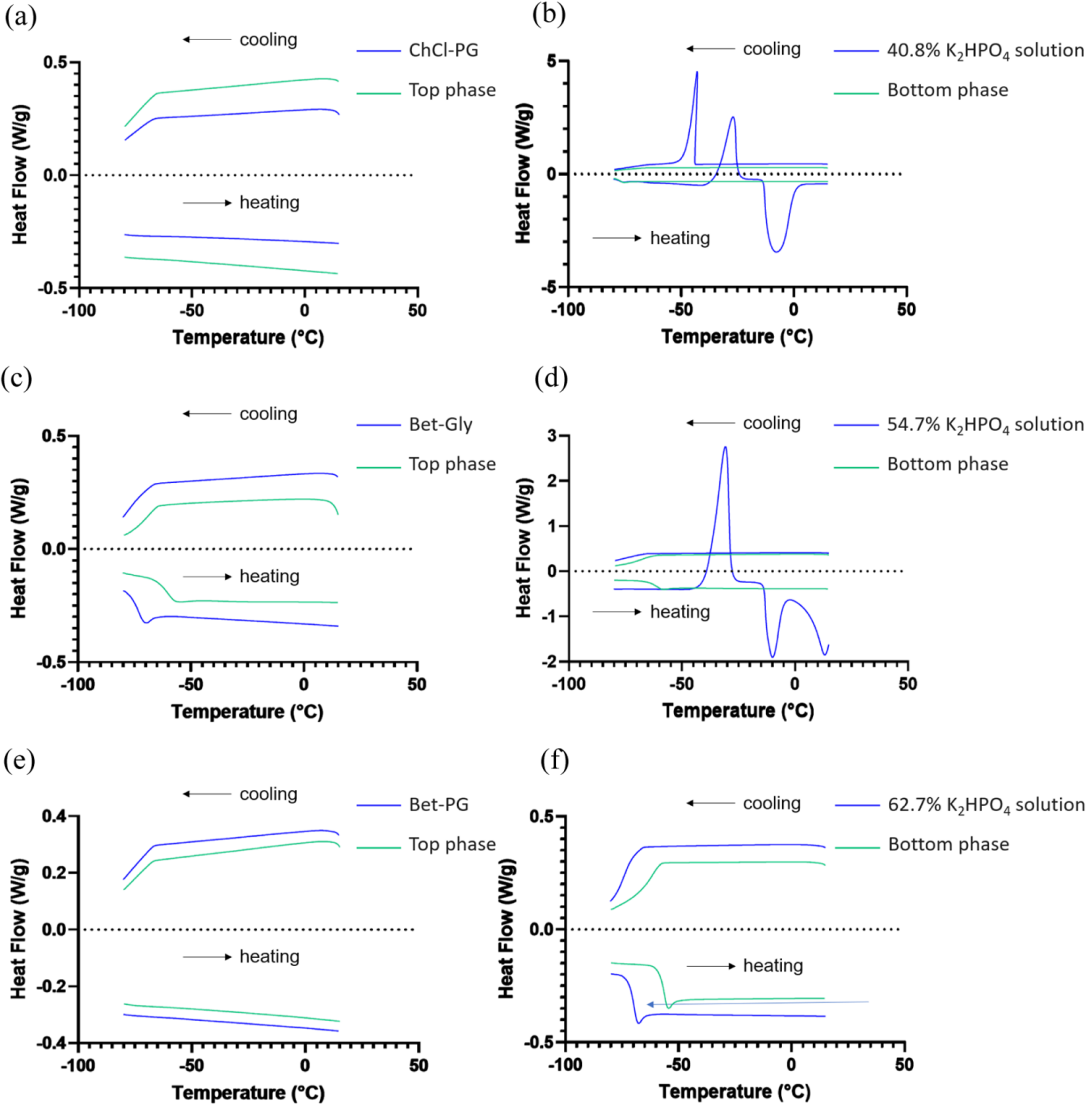
Heat flow thermograms obtained at 10 °C/min for: (a) ChCl:PG and the top phase of ChCl:PG-K_2_HPO_4_ ATPS, (b) 40.8 wt % K_2_HPO_4_solution and the bottom phase of ChCl:PG-K_2_HPO_4_ ATPS, (c) Bet:Gly and the top phase of Bet:Gly-K_2_HPO_4_ ATPS, (d) 54.7 wt % K_2_HPO_4_ solution and the bottom phase of Bet:Gly-K_2_HPO_4_ ATPS, (e) Bet:PG and the top phase of Bet:PG-K_2_HPO_4_ ATPS, and (f) 62.7 wt % K_2_HPO_4_ solution and the bottom phase of Bet:PG-K_2_HPO_4_ ATPS.

Similar to Bet:Gly, which had a glass transition temperature of −74.2 °C (Figure 3g), the top phase of Bet:Gly-K_2_HPO_4_ ATPS exhibited a glass transition, though at a higher temperature (−61.4 °C) (Figure 4c). While the inclusion of water typically lowers the *T*_g_ value (since pure water has a glass transition temperature of about −137 °C), several factors in this system may counterbalance or even reverse this effect.^40^ The K_2_HPO_4_ present in the top phase (31.98% of the salt) (Table 3) might contribute to the observed higher *T*_g_ value. Besides, the salt’s strong water-binding capacity could further increase *T*_g_. Similarly, betaine zwitterions and glycerol molecules reduce the amount of free water, raising the *T*_g_ value.^41^

Upon cooling and heating of K_2_HPO_4_ solutions of 40.8 and 54.7 wt %, crystallization and melting events were observed (Figure 4b,d). In contrast, no crystallization and melting were detected for the bottom phase of the ChCl:PG-K_2_HPO_4_ ATPS and Bet:Gly-K_2_HPO_4_ ATPS. This discrepancy can be attributed to the differences in water content. The NADES used in this study contained no water, with water introduced only via the K_2_HPO_4_ solutions. During phase separation, the NADES and K_2_HPO_4_ competed over water molecules, resulting in a lower water content in the bottom phase (at equilibrium), compared to the initial 40.8 wt % K_2_HPO_4_ solution. At low water content, interactions between water and K_2_HPO_4_ molecules become more prominent, significantly affecting the thermal behavior.

As shown in Figure 4f, a glass transition was observed at −70.8 °C for the K_2_HPO_4_ solution of 62.7 wt % and at −57.6 °C for the bottom phase of the Bet:PG-K_2_HPO_4_ ATPS, likely due to the high K_2_HPO_4_ concentration in both samples. The 62.7 wt % K_2_HPO_4_ solution contained approximately 6165 mM dipotassium phosphate, while the concentration in the bottom phase of the Bet:PG-K_2_HPO_4_ ATPS was even higher, at 7449 mM (Table 3). Similarly, a K_2_HPO_4_-saturated aqueous solution has been reported to enter a glassy state without crystallization at about −73 °C.^42^

The top and bottom phases of the NADES-K_2_HPO_4_ ATPSs exhibited either a single glass transition or no thermal events, indicating their homogeneity. This homogeneity along with their stability across a broad temperature range without crystallization suggests that the structure of NADES is preserved in the top phase. Consequently, NADES-K_2_HPO_4_ ATPS may be promising candidates for use as cryoprotective media.

### Partition Behavior

Since the addition of alanine, phenylalanine, and glucose to the Bet:Gly-K_2_HPO_4_ ATPS resulted in the formation of a single-phase solution, the partition behavior analysis was conducted using ChCl:PG-K_2_HPO_4_ ATPS and Bet:PG-K_2_HPO_4_ ATPS. As reported in Table 7, glucose exhibited very low mean *K* values of 0.08 in ChCl:PG-K_2_HPO_4_ATPS and 0.03 in Bet:PG-K_2_HPO_4_ ATPS. In contrast, alanine displayed mean *K* values of 2.27 and 10.35 in these two ATPSs, respectively. Notably, phenylalanine was undetectable in the bottom phases of both ATPSs, indicating that nearly 100% of the phenylalanine partitioned into the top phase. These results suggest that glucose has an affinity to the bottom phase, whereas alanine and phenylalanine are preferentially partitioned into the top phase (Table 7). These findings align with the octanol–water partition coefficients of the studied molecules: −3.10 for glucose,^43^ −2.85 for alanine,^44^ and −1.38 for phenylalanine.^45^ Higher partition coefficients reflect greater hydrophobicity, with phenylalanine exhibiting the highest values among the molecules studied. Furthermore, both Bet:PG and ChCl:PG are hydrophobic NADESs, making the top phase more hydrophobic than the bottom phase.^35^ The highly hydrophobic top phase attracted phenylalanine more strongly than did alanine and glucose. Compared to previous reports, such as Farias et al.,^46^ where phenylalanine exhibited K values below 25 in ChCl:PG-K_2_HPO_4_ ATPSs, our systems showed an even stronger enrichment in the NADES phase. This significantly higher partitioning efficiency may be attributed to differences in phase composition, particularly the higher wt % of NADES content in our systems. Specifically, the ChCl:PG-K_2_HPO_4_ ATPS used 43.6 wt % ChCl:PG and 23.0 wt % K_2_HPO_4_, while the Bet:PG-K_2_HPO_4_ ATPS contained 39.2 wt % Bet:PG and 38.1 wt % K_2_HPO_4_. In contrast, the system reported by Farias et al. used only 20 wt % ChCl:PG and 40 wt % K_2_HPO_4_. The significantly higher NADES content in our systems enhanced the hydrophobicity of the top phase, thereby increasing its affinity for hydrophobic solutes, such as phenylalanine. These results indicate the high potential of Bet:PG-*K*_2_ *HPO*_4_ ATPS and ChCl:PG-K_2_HPO_4_ ATPS in the partitioning of glucose, alanine, and phenylalanine.

**Table 7:** Partition Coefficient (*K*) and Partition Extent at the Top Phase (*E*_top_) of Glucose, Alanine, and Phenylalanine in ChCl:PG–K_2_HPO_4_ and Bet:PG-K_2_HPO_4_

ATPS	molecules	concentration in the top phase (mM)	concentration in the bottom phase (mM)	*K*	*E*_top_ (%)
ChCl:PG-K_2_HPO_4_	glucose	10.49 ± 0.26	139.49 ± 7.65	0.08 ± 0.00	18.41 ± 0.07
	alanine	50.20 ± 0.90	22.20 ± 1.28	2.27 ± 0.17	87.32 ± 0.61
	phenylalanine	50.00 ± 2.00	<0.14	>357.16	∼ 100
Bet:PG-K_2_HPO_4_	glucose	2.65 ± 0.10	83.80 ± 8.92	0.03 ± 0.01	4.02 ± 0.37
	alanine	58.62 ± 1.02	5.66 ± 0.88	10.35 ± 1.03	93.21 ± 0.51
	phenylalanine	61.10 ± 5.14	<0.14	>436.44	∼ 100

### The Development of the Maillard Reaction

Bet:PG-K_2_HPO_4_ ATPS was chosen for the Maillard reaction because of its superior ability to partition glucose, alanine, and phenylalanine (Table 7). Epifluorescence microscopy imaging was used to identify the dispersed and continuous phases in the Bet:PG-K_2_HPO_4_ ATPS. As expected, due to the lower volume of the NADES-rich phase, the emulsion was Bet:PG-in-K_2_HPO_4_ (Figure 5). The droplet diameter ranged from several tens of nanometers to over 100 μm, reflecting that the droplets coalesced during sample preparation for microscopic imaging. It is worth noting that throughout the Maillard reaction, the ATPS samples were continuously stirred, preventing droplet coalescence.

**Figure 5:**
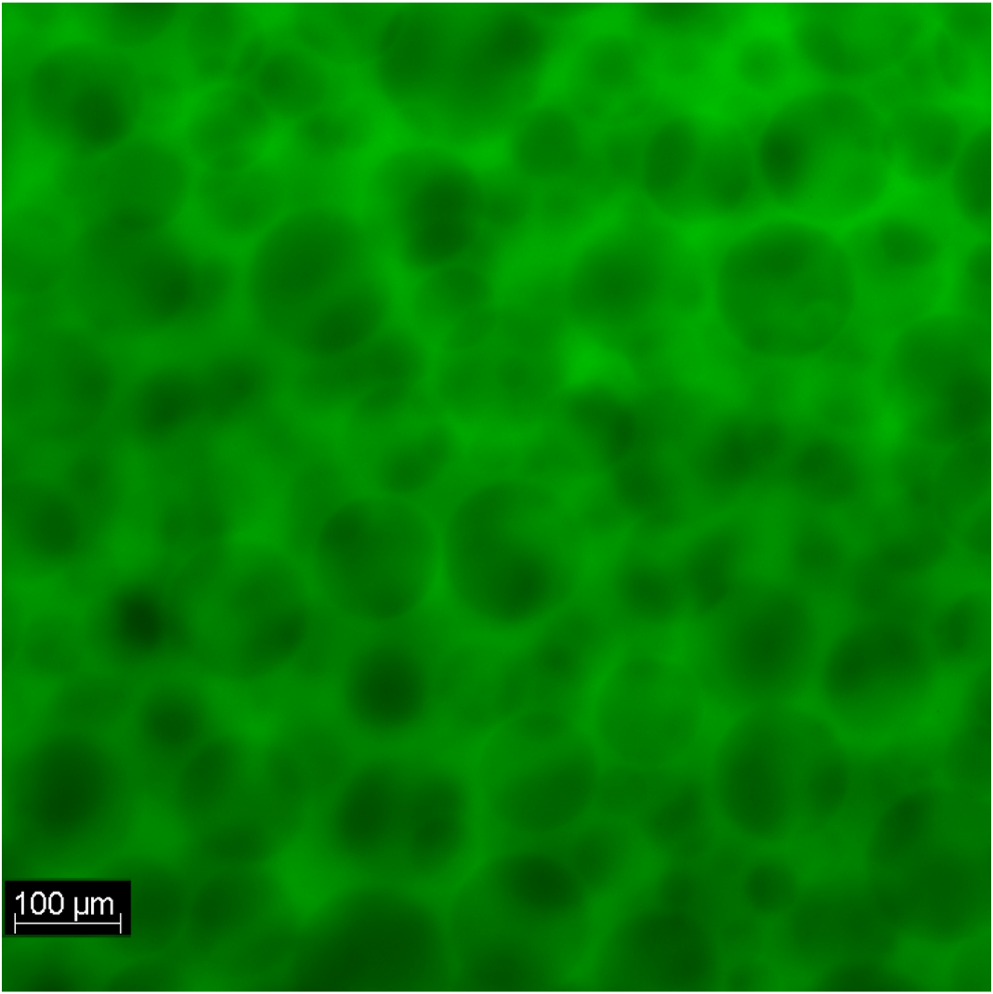
Representative epi-fluorescent microscopy image of Bet:PG-K_2_HPO_4_ ATPS scaled at 100 μm. FITC accumulates in the Bet:PG-rich phase, indicating that it is a continuous phase.

The initial stage of the Maillard reaction between glucose and alanine/phenylalanine was used as a model to explore the application of Bet:PG-K_2_HPO_4_ ATPS. Amadori products formation in ATPS samples and control solutions at 25 and 37 °C during a 5-day period was monitored as a reference to test the ability of NADES-K_2_HPO_4_ ATPSs for modulating the location of precursors, their condensation, and the rearrangement of intermediates and products. Figure 6 presents the concentration of reactants and the yield of Amadori compounds in whole ATPS, Top-priorPS, Bottom-priorPS, Top-postPS, and Bottom-postPS. Glucose concentration in whole ATPS, Bottom-priorPS, and Top-postPS significantly decreased over the incubation period (Figure 6a,b). Similarly, the amino acid concentration in whole ATPS, Top-priorPS, and Top-postPS showed a significant decline with incubation time (Figure 6c,d) even at 37 °C. Notably, the concentration of phenylalanine in Bottom-postPS at day 0 was only 4.52 mM (Figure 6c), significantly lower than the initial 40 mM, thus suggesting a diversified array of reactions. This reduction was attributed to the high concentration of potassium dihydrogen phosphate in the bottom phase, which decreases the solubility of phenylalanine.

**Figure 6:**
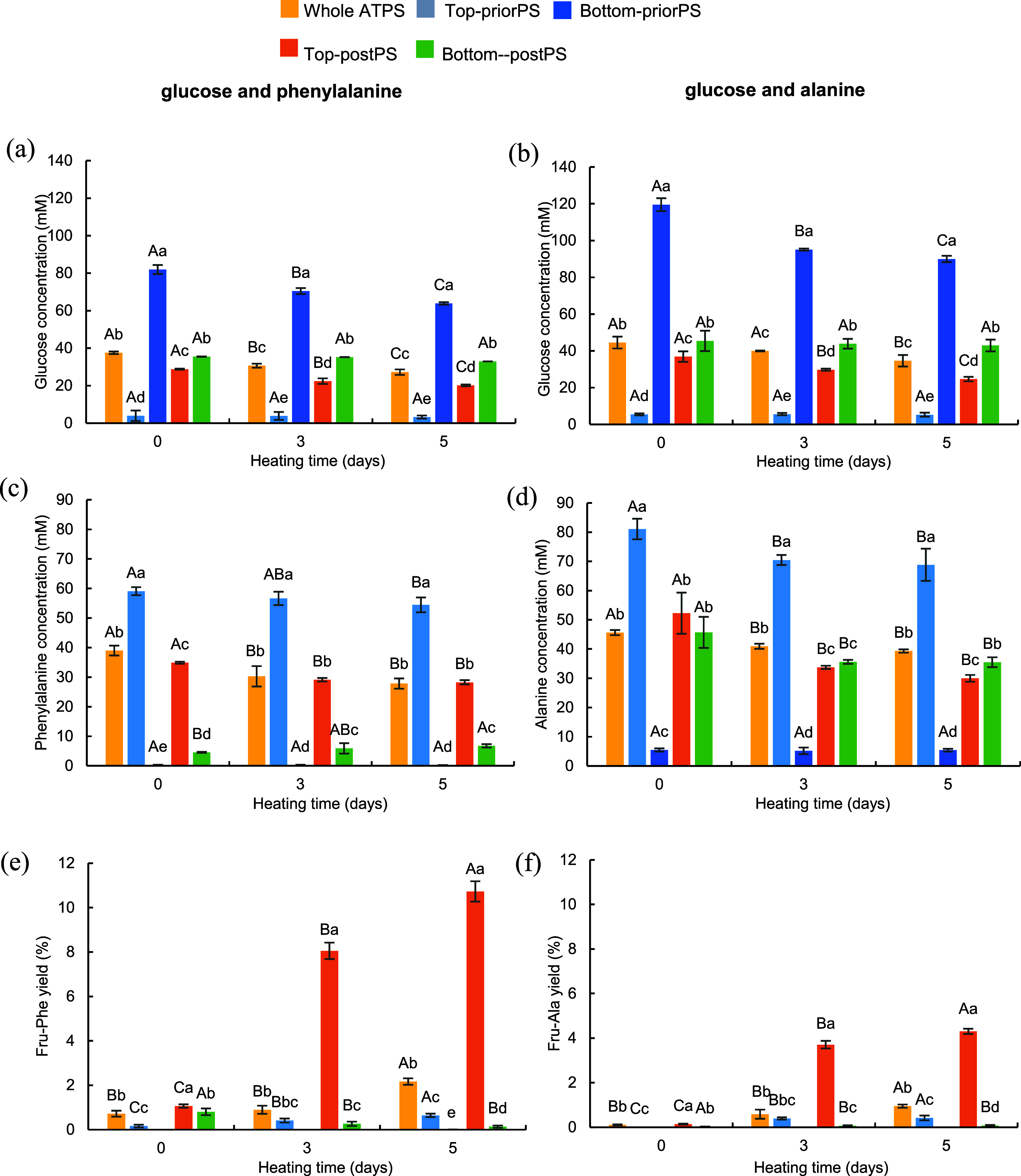
Concentration of glucose (a, b), phenylalanine (c), alanine (d), and the yield of the corresponding Amadori compounds (e, f) in whole ATPS, Top-priorPS, Bottom-priorPS, Top-postPS, and Bottom-postPS after 0, 3, and 5 days of incubation at 37 °C. The concentration of glucose, phenylalanine, and alanine used in the Maillard reaction was 40 mM. Different lowercase letters indicate significant differences between samples at the same time point (*P* < 0.05). Different uppercase letters indicate significant differences between samples across different heating times (*P* < 0.05).

As shown in Figure 6e, Fru-Phe formation (yield 0.80% relative to the amount of amino acid) was observed in Bottom-postPS at day 0, despite the incomplete dissolution of phenylalanine. The yield declined sharply to 0.14% on day 5. In the control samples, with reactants dissolved in water, the reduction of the two amino acids and glucose was negligible, and no Amadori compounds were detected on days 3 and 5 (data not shown). The difference between the control and Bottom-postPS suggests that a high concentration of potassium dihydrogen phosphate may facilitate Fru-Phe accumulation at room temperature.

In Top-postPS (Figure 6e,f), Amadori compounds were already present at day 0, and their yield continued to increase over time. By day 5, Fru-Phe and Fru-Ala yields in Top-postPS reached 10.73 and 4.30%, respectively. These results indicate that NADES significantly promotes Amadori compound accumulation at both 25 and 37 °C. Betaine, with its carboxylate group (−COO^–^) containing two negatively charged oxygen atoms, acts as a strong hydrogen bond acceptor.^47^ It forms hydrogen bonds with the hydroxyl groups of the fructose moiety in the Amadori compounds, creating a network that restricts their molecular freedom and reduces their likelihood of undergoing retro-aldol cleavage, which would lead to the formation of fragmentation products. Additionally, propylene glycol, which contains two adjacent hydroxyl groups as hydrogen bond donors (HBD), interacts with the fructosyl moiety hydroxyl groups, further stabilizing the molecular structures of the Amadori compounds, thus reducing their degradation. Furthermore, the viscosity of NADES plays a crucial role. Su, Yu, Wang, Zhao, Zhao, and Zhang utilized a NADES system composed of proline, glucose, and water to conduct the Maillard reaction at 80 °C.^48^ They found that when the water content was 15%, the yield of Amadori compounds was several times higher than those in a conventional aqueous system. The high viscosity of NADES likely limits the diffusion and the mobility of the Amadori compounds, reducing their collisions with other reactants and thereby inhibiting subsequent chemical reactions.^49^ The yield of Amadori compounds in whole ATPS remained significantly lower than that in Top-postPS (Figure 6e,f). This is because amino acids in ATPS primarily partitioned into the NADES-rich phase (Figure 6c,d), while glucose preferentially segregated into the K_2_HPO_4_-rich phase (Figure 6a,b). Unlike single-phase solutions, ATPSs facilitate reactions by confining partitioned reactants to the water–water interface, where the reactants encounter. However, this restricted interaction limits the promoting effect of NADES on the reaction, thereby slowing the accumulation of Amadori compounds. In addition, Figure 6e,f shows that the Amadori compounds are partitioned within the NADES-rich phase, colocalizing with amino acids. This suggests that their hydrophobicity is like that of amino acids, enabling ATPS to preseparate the Amadori products. The phase separation behavior of ATPSs could be leveraged for the selective extraction and purification of Amadori compounds, reducing the need for additional downstream processing. Furthermore, the compartmentalization of reactants in different phases may enable controlled reaction kinetics, potentially minimizing unwanted side reactions and degradation pathways.

## Conclusions

The characterization of selected NADESs (ChCl:Gly, ChCl:PG, Bet:Gly, and Bet:PG) and their interactions with K_2_HPO_4_ provided critical insights into their physicochemical properties, phase behavior, and potential applications. ^1^H NMR and DSC analyses confirmed the formation and stability of these NADESs, with molar ratios consistent with their preparation. Their thermal stability across a wide temperature range supports their versatility as solvents. The formation of NADES-K_2_HPO_4_ ATPS was strongly influenced by the hydrophilicity of NADES components, with Bet:PG exhibiting the highest hydrophobicity and requiring the lowest K_2_HPO_4_ concentration for phase separation. Key physical properties, including density, viscosity, pH, and water activity, further demonstrated how molecular composition and water interactions shape system characteristics. DSC results also confirmed the structural integrity of NADESs in the top phases. Partitioning studies highlighted distinct behaviors of glucose, alanine, and phenylalanine in Bet:PG-K_2_HPO_4_ and ChCl:PG-K_2_HPO_4_ ATPSs. Glucose, with low hydrophobicity, preferentially partitioned into the bottom phase, whereas alanine and phenylalanine partitioned into the hydrophobic top phases. Phenylalanine, the most hydrophobic, was almost entirely confined to the top phase. Finally, this study demonstrated the role of NADES in promoting the formation and stabilization of Amadori compounds. The compartmentalization of reactants in emulsions further highlighted the advantages of Bet:PG-K_2_HPO_4_ ATPS for controlled reaction kinetics and product separation. Future work will focus on optimizing the system to maximize amino acid partitioning-extraction efficiency and further enhance selectivity through fine-tuning of the NADES composition and salt concentrations, thereby advancing their applicability in industrial-scale separation and reaction systems.

Overall, these findings underscore the unique properties of NADES-K_2_HPO_4_ ATPS systems, including their ability to maintain a stable supramolecular network and facilitate selective biomolecule separation and Amadori compounds formation. These results enhance our understanding of NADES-based ATPSs and highlight their potential for applications in biochemistry, food science, and green chemistry. It should be noted, however, that this study only monitored Amadori compounds as early-stage Maillard reaction products, without tracking downstream products such as advanced glycation end-products, which limits a comprehensive understanding of the role of NADES in the entire Maillard reaction pathway.

## Supplementary Material



## Notes

^1^H, ^13^C, and HSQC spectra of individual NADES components; ^13^C, HSQC, and COSY spectra of synthesized NADESs; and ^1^H NMR spectra of NADES- K_2_HPO_4_ ATPS phases (PDF)
