# Supplementary material for: Natural Deep Eutectic Solvent–Dipotassium Phosphate Aqueous Two-Phase Systems: Physicochemical Characterization, Selective Partitioning of Amino Acids and Glucose, and Functional Insight into Maillard Reaction Applications
Source: ACS Sustain Chem Eng. 2025 Jul 21;13(30):11898–912. doi: 10.1021/acssuschemeng.5c03053 (PMC12327354; doi:10.1021/acssuschemeng.5c03053)
Supplement: Supplementary file 1 [file sc5c03053_si_001.pdf]

Supporting information

Natural Deep Eutectic Solvent - Dipotassium Phosphate Aqueous Two-Phase Systems: Physicochemical Characterization, Selective Partitioning of Amino Acids and Glucose, and Functional Insight into Maillard Reaction Applications

Kangni Chen [a], Antonio Dario Troise [b], Anton Bunschoten [c], Sabrina De Pascale [b], Andrea Scaloni [b], Vincenzo Fogliano [a], Ashkan Madadlou [d, *]

[a] Food Quality and Design Group, Wageningen University & Research, 6708WG Wageningen, The Netherlands

[b] Proteomics, Metabolomics & Mass Spectrometry Laboratory, ISPAAM, National Research Council, 80055 Portici, Italy

[c] BioNanoTechnology, Wageningen University and Research, 6708WG Wageningen, The Netherlands

[d] School of Food and Nutritional Sciences, University College Cork (UCC), Cork T12 Y337, Ireland

This supplemental document contains 15 figures in 12 pages.

Supplementary Figures

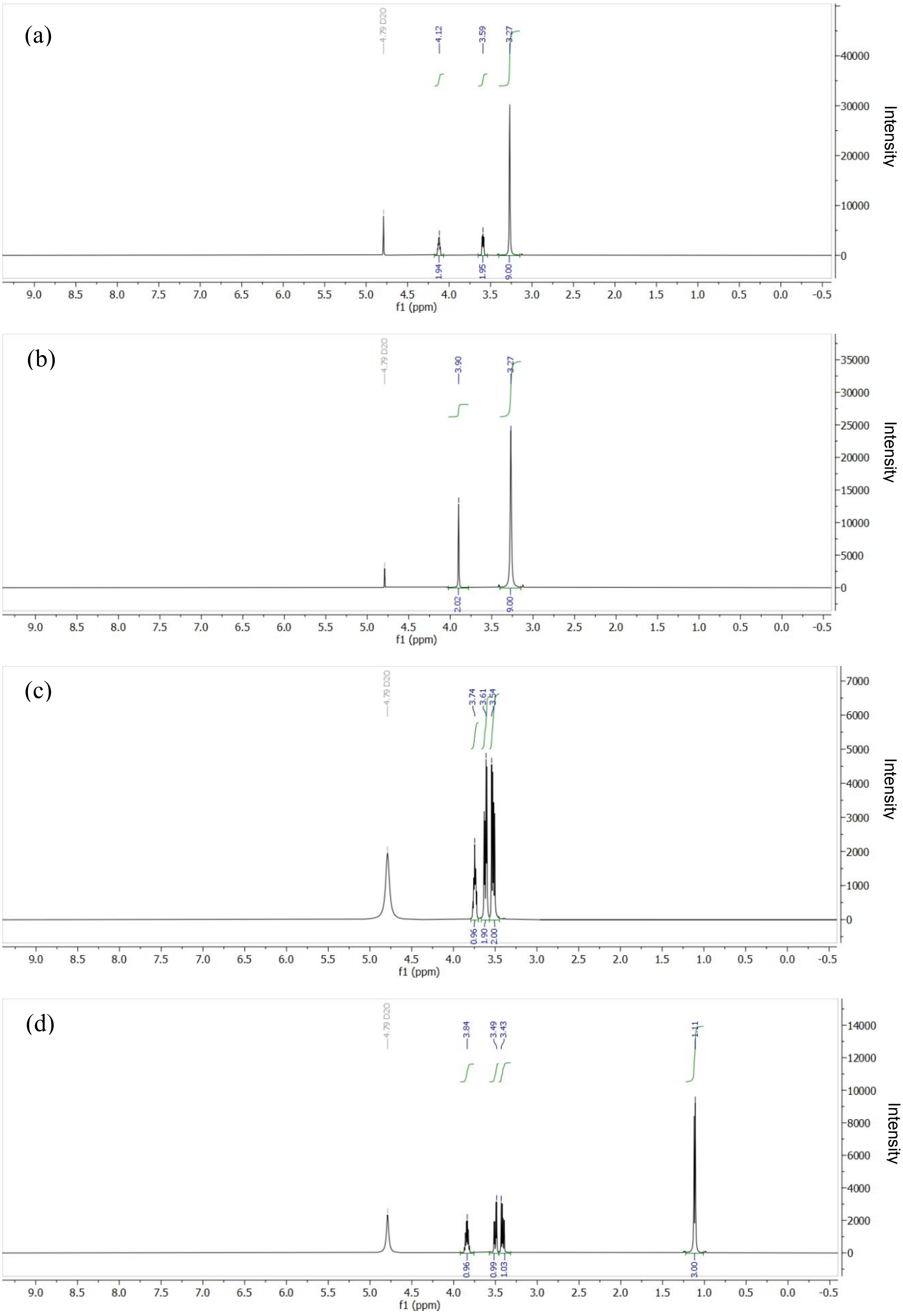

Figure S1. The ^{1}H NMR spectra of (a) choline chloride, (b) betaine, (c) glycerol, and (d) propylene glycol.

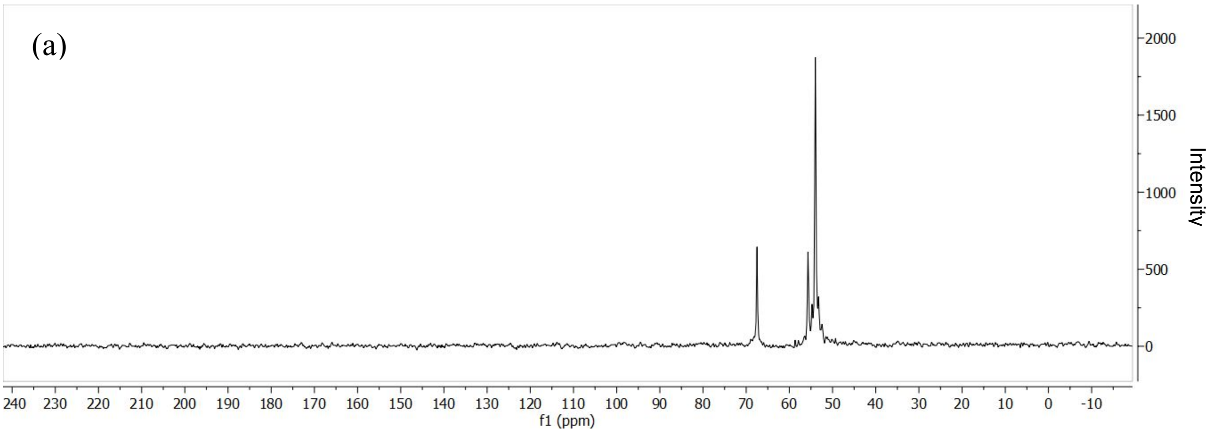
(a)
Intensity
f1 (ppm)

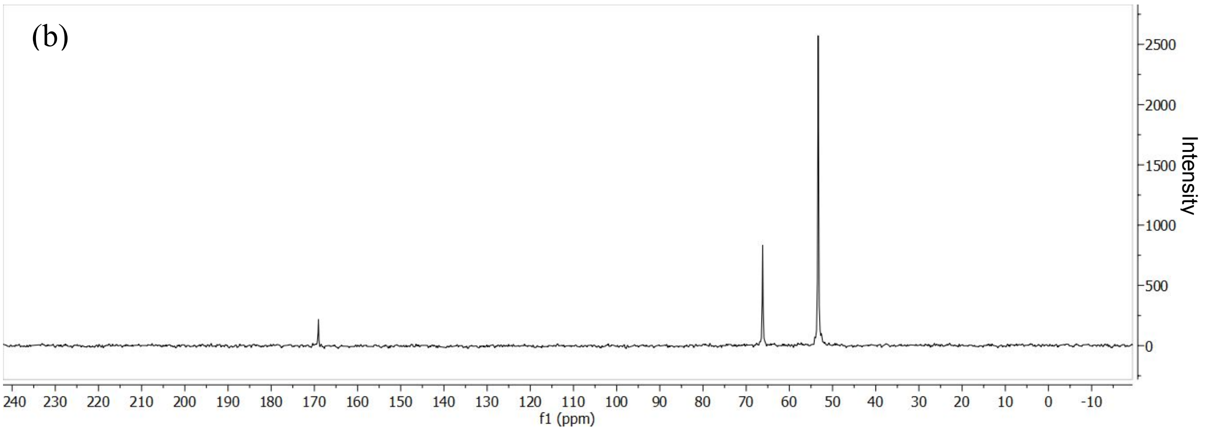
(b)
Intensity
f1 (ppm)

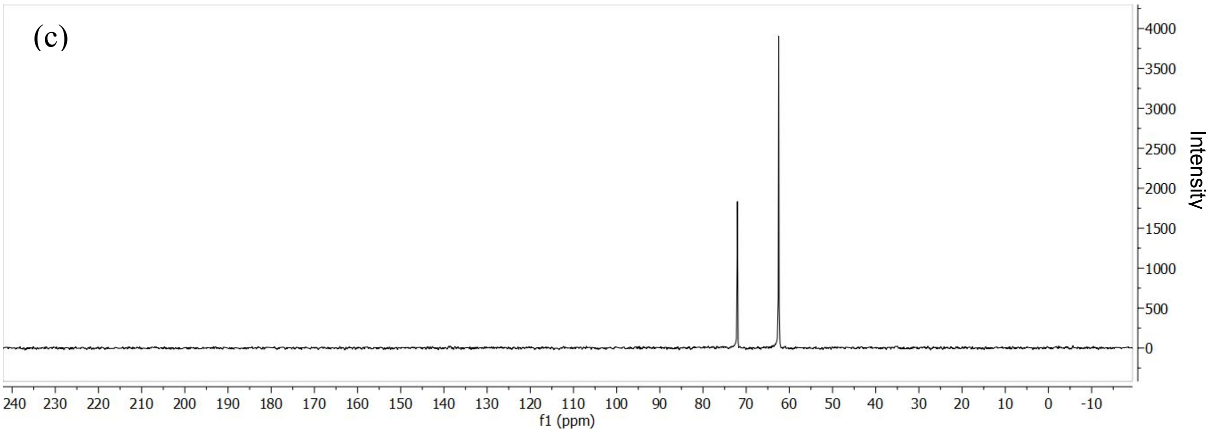
(c)
Intensity
f1 (ppm)

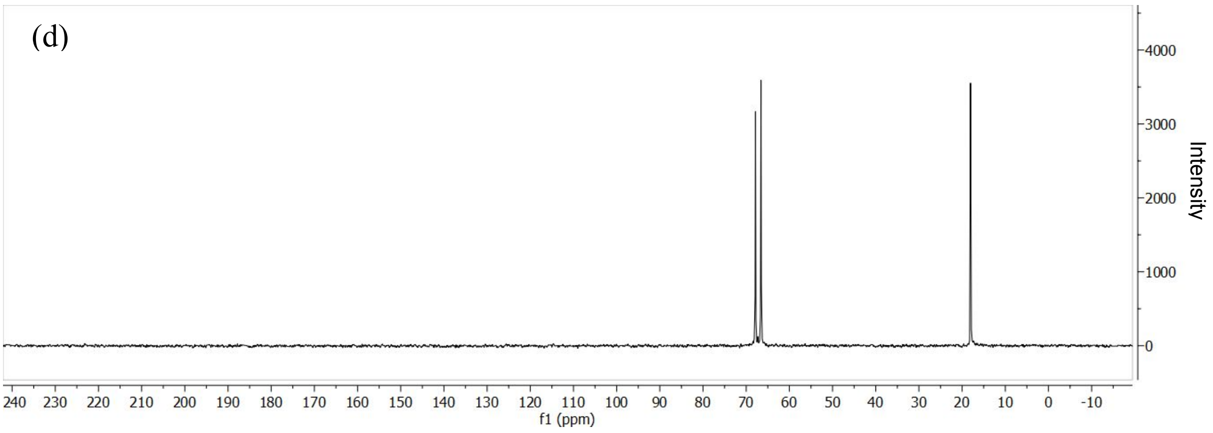
(d)
Intensity
f1 (ppm)

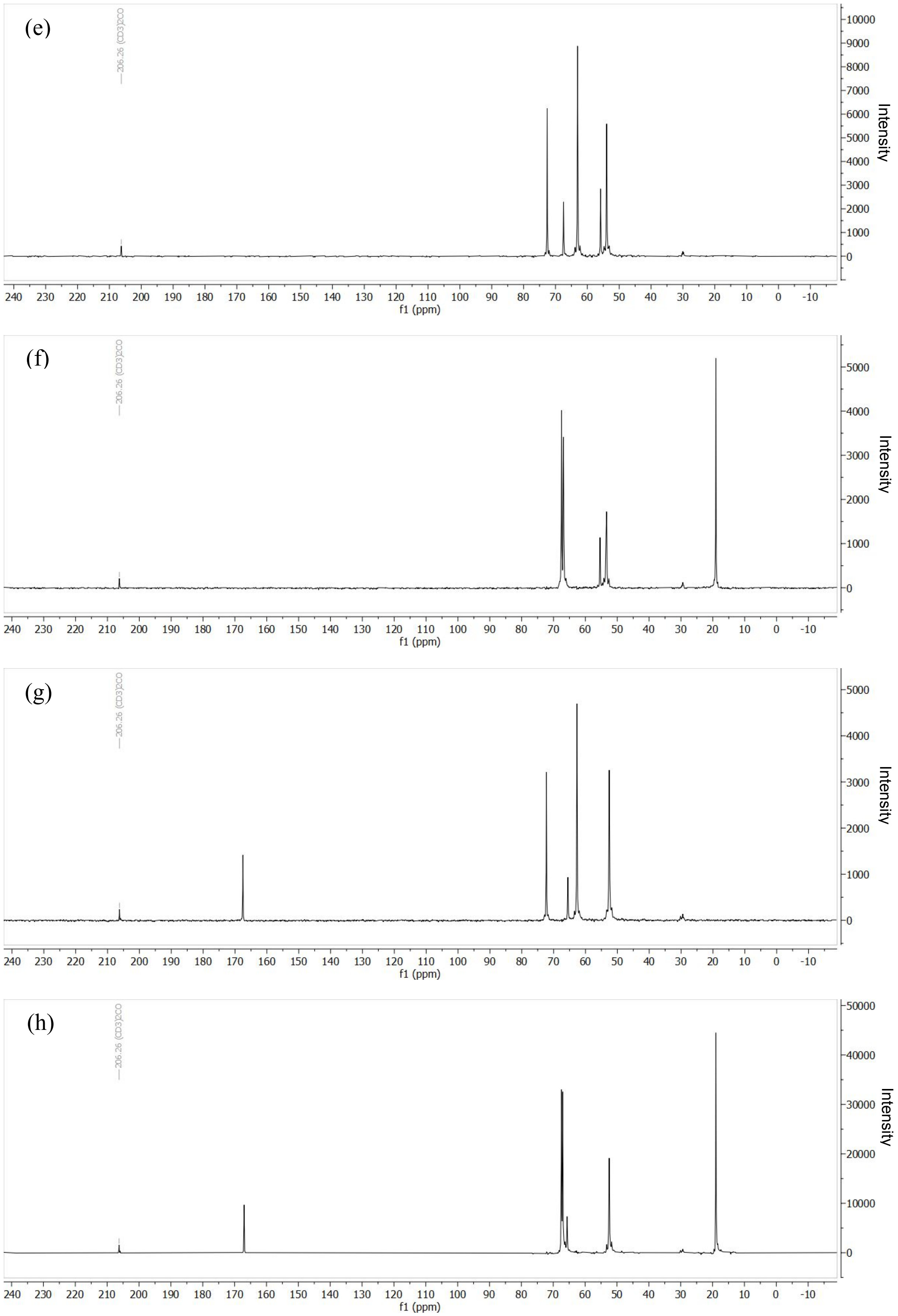

Figure S2. The ^{13}C NMR spectra of (a) choline chloride, (b) betaine, (c) glycerol, (d) propylene glycol, (e) ChCl:Gly, (f) ChCl:PG, (g) Bet:Gly, and (h) Bet:PG.

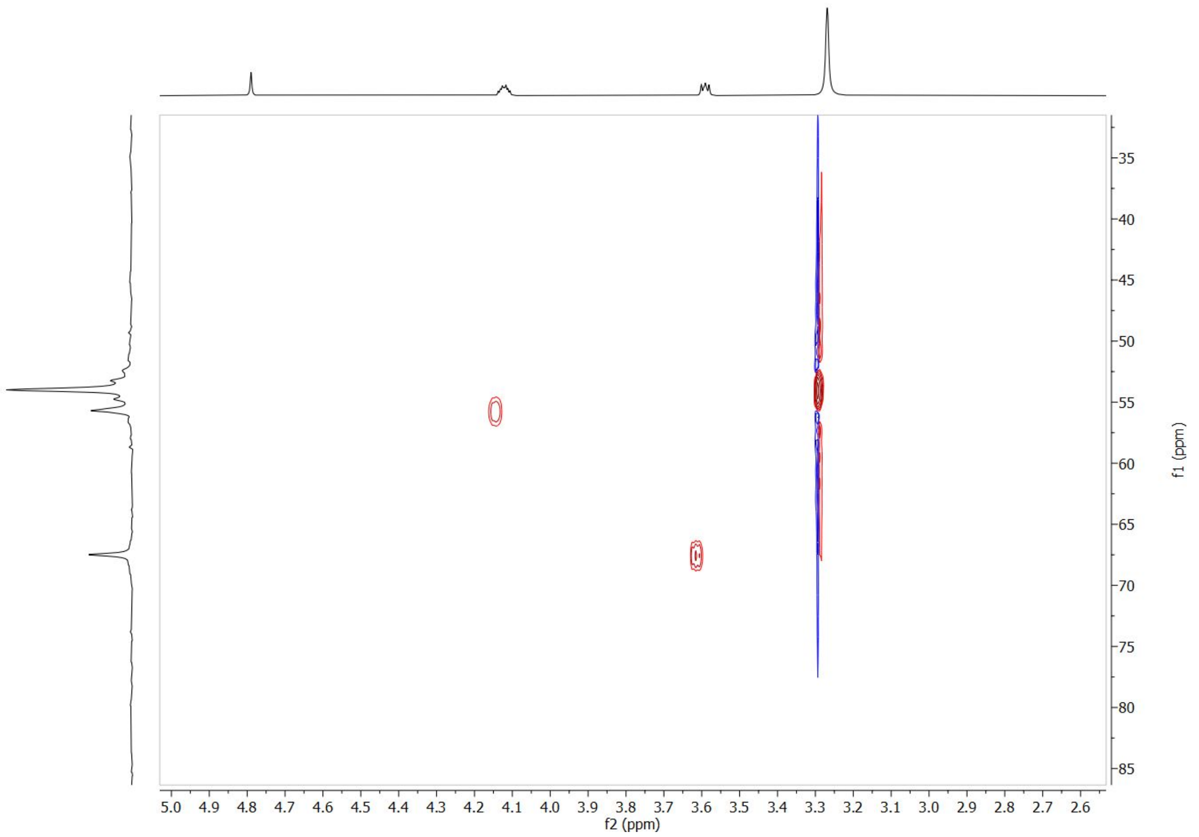

Figure S3. ^{1}H-^{13}C HSQC spectrum of choline chloride.

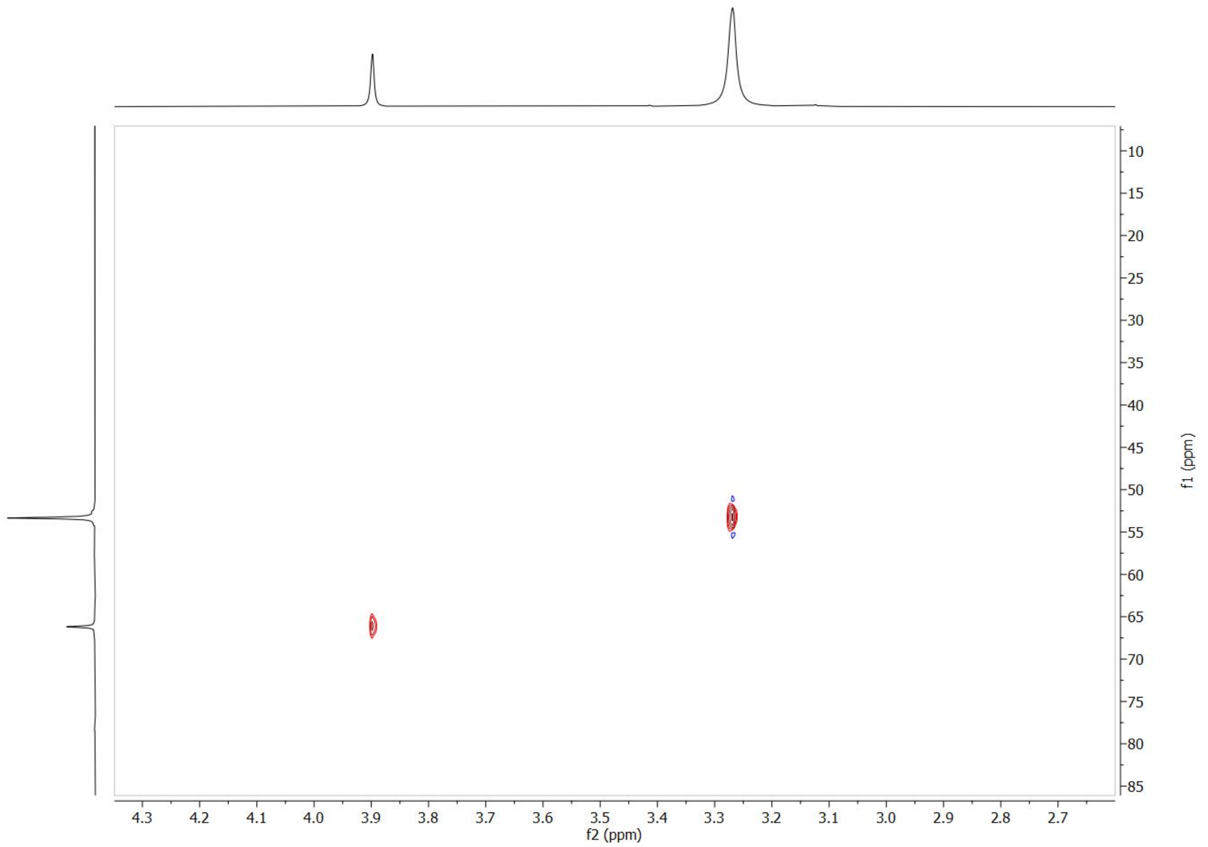

Figure S4. ^{1}H-^{13}C HSQC spectrum of betaine.

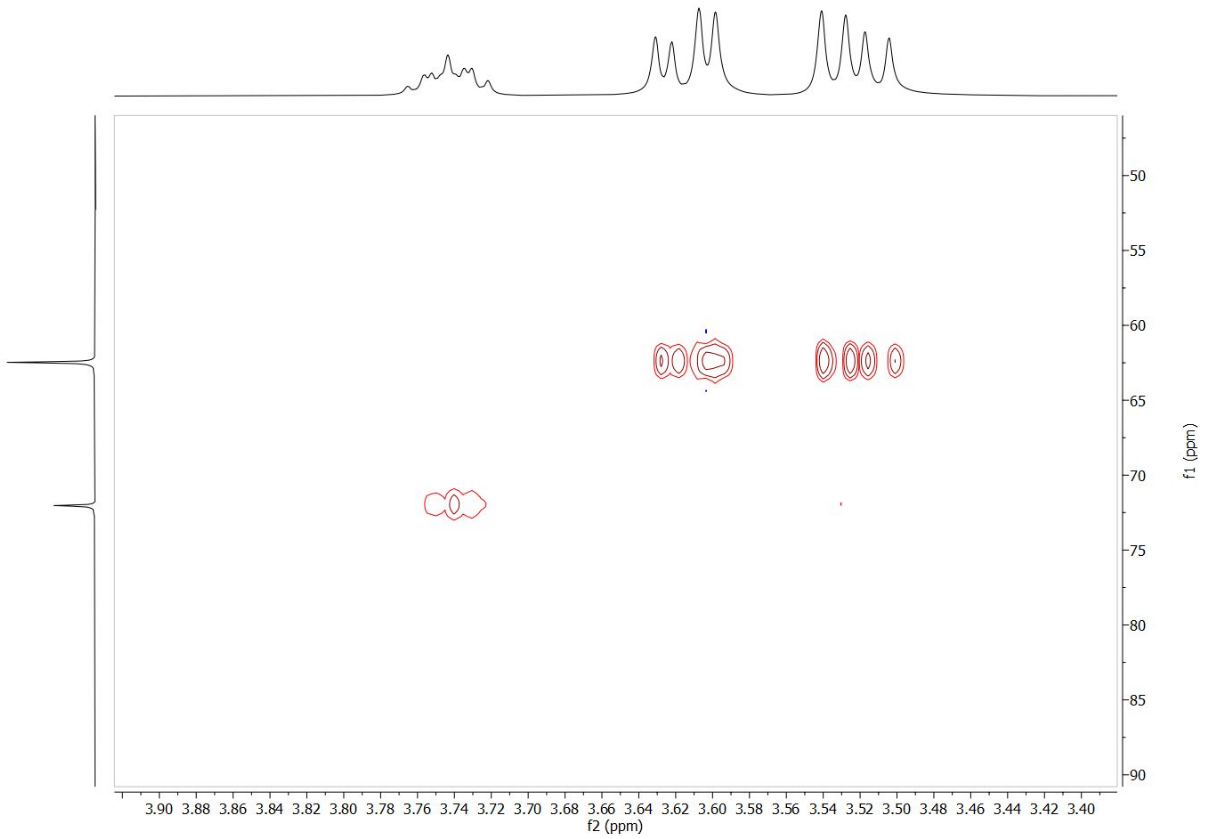

Figure S5. ^{1}H-^{13}C HSQC spectrum of glycerol.

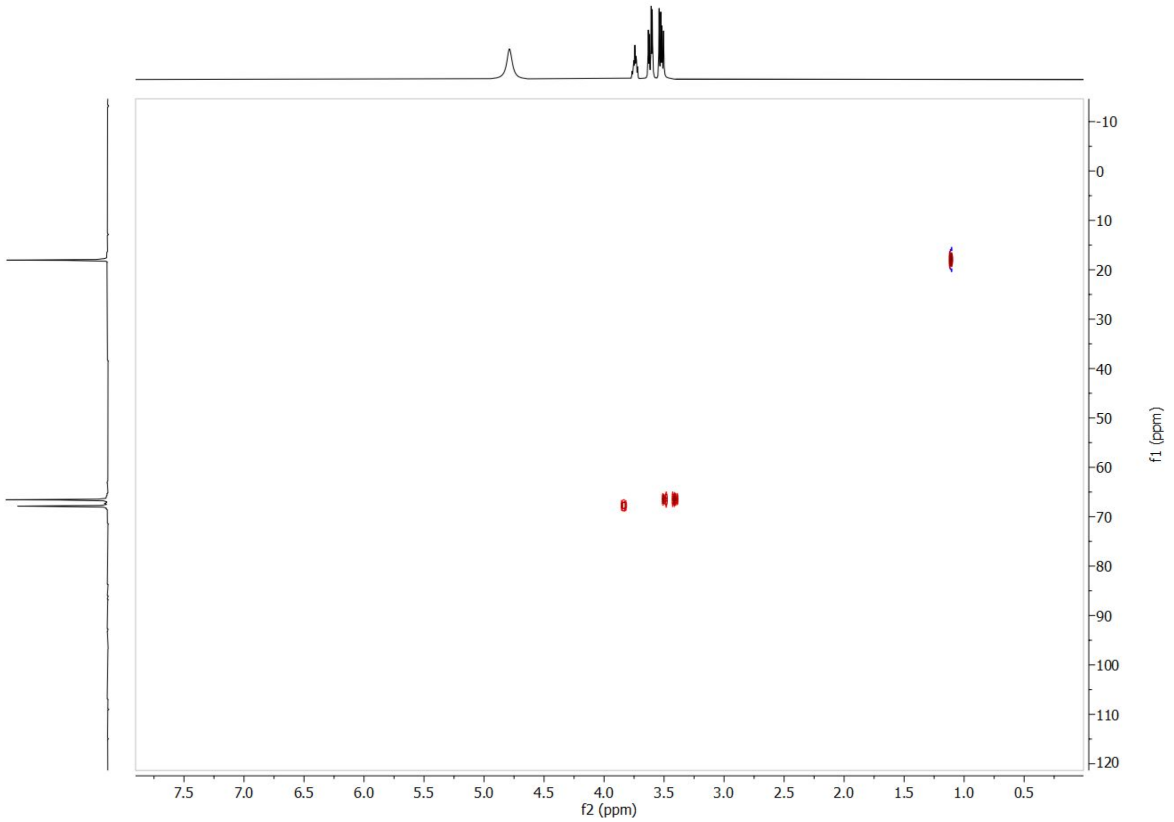

Figure S6. ^{1}H-^{13}C HSQC spectrum of polypropylene glycol.

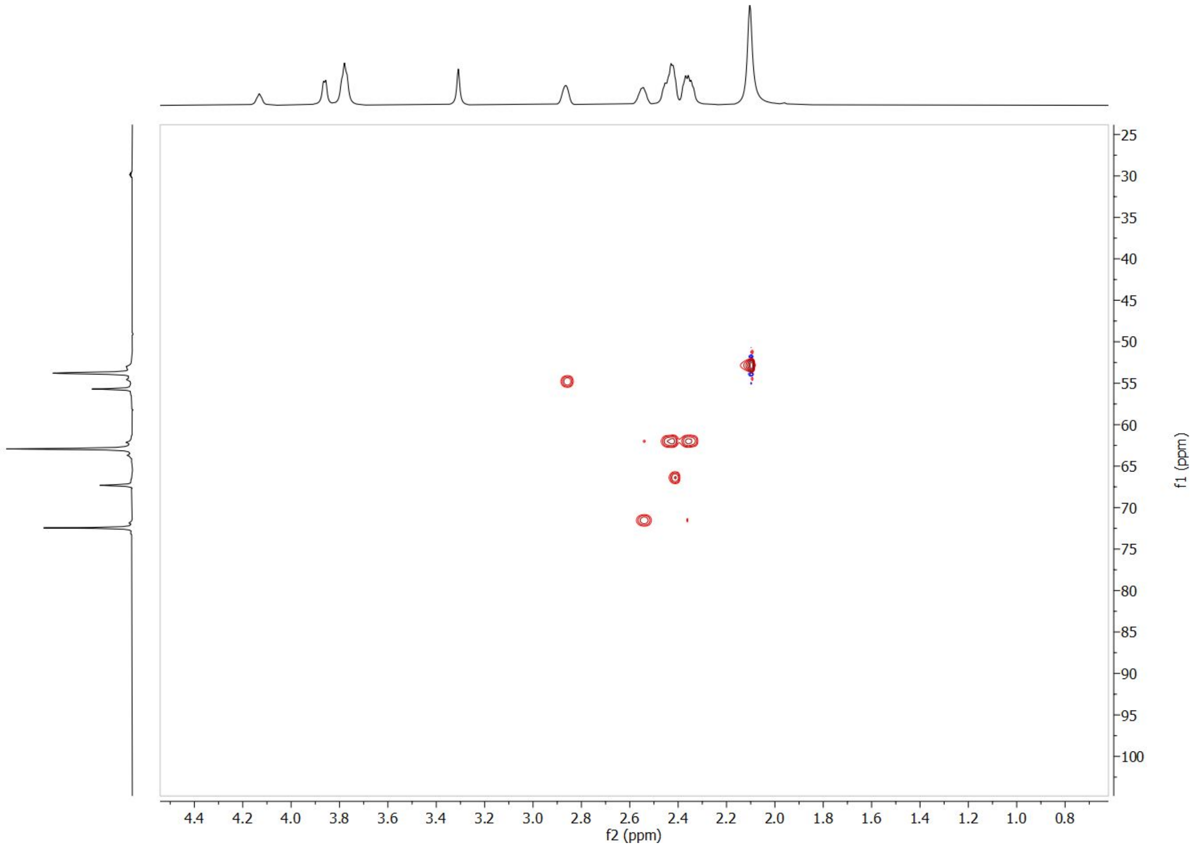

Figure S7. ^{1}H-^{13}C HSQC spectrum of ChCl:Gly.

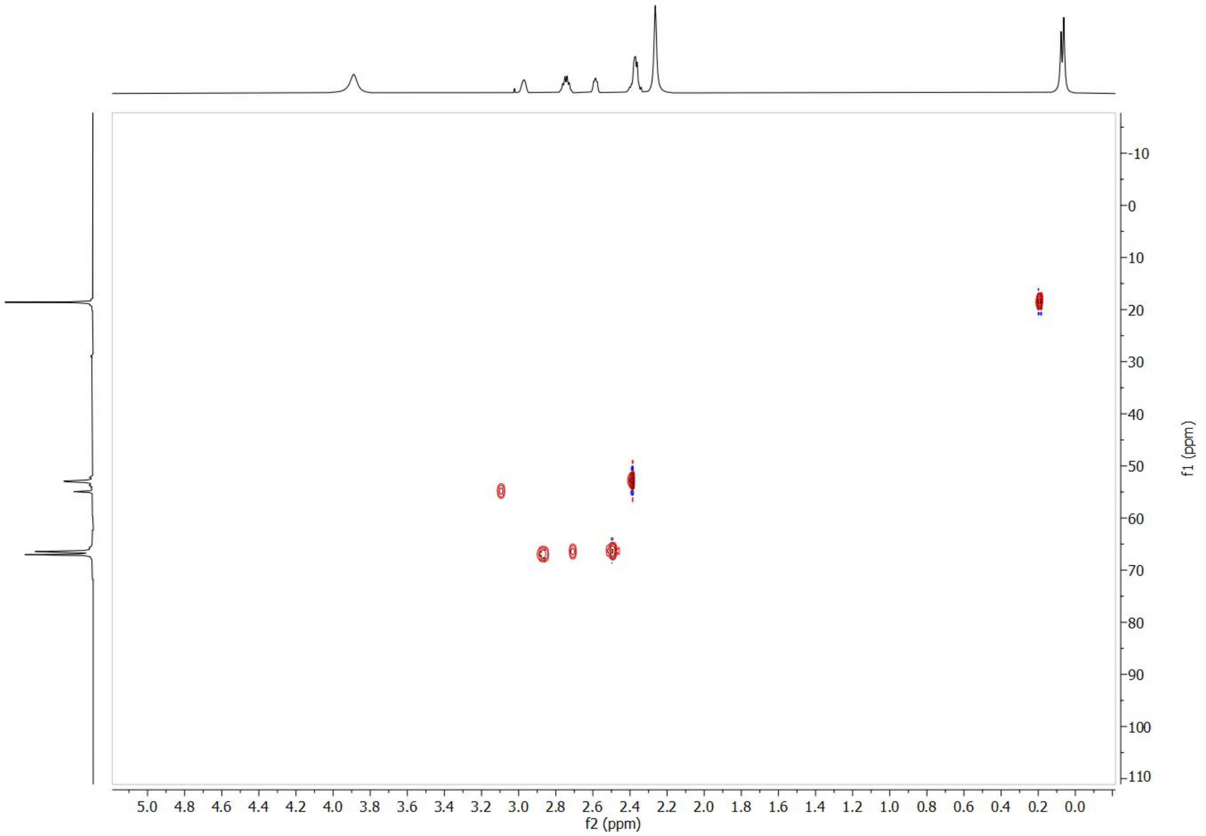

Figure S8. ^{1}H-^{13}C HSQC spectrum of ChCl:PG.

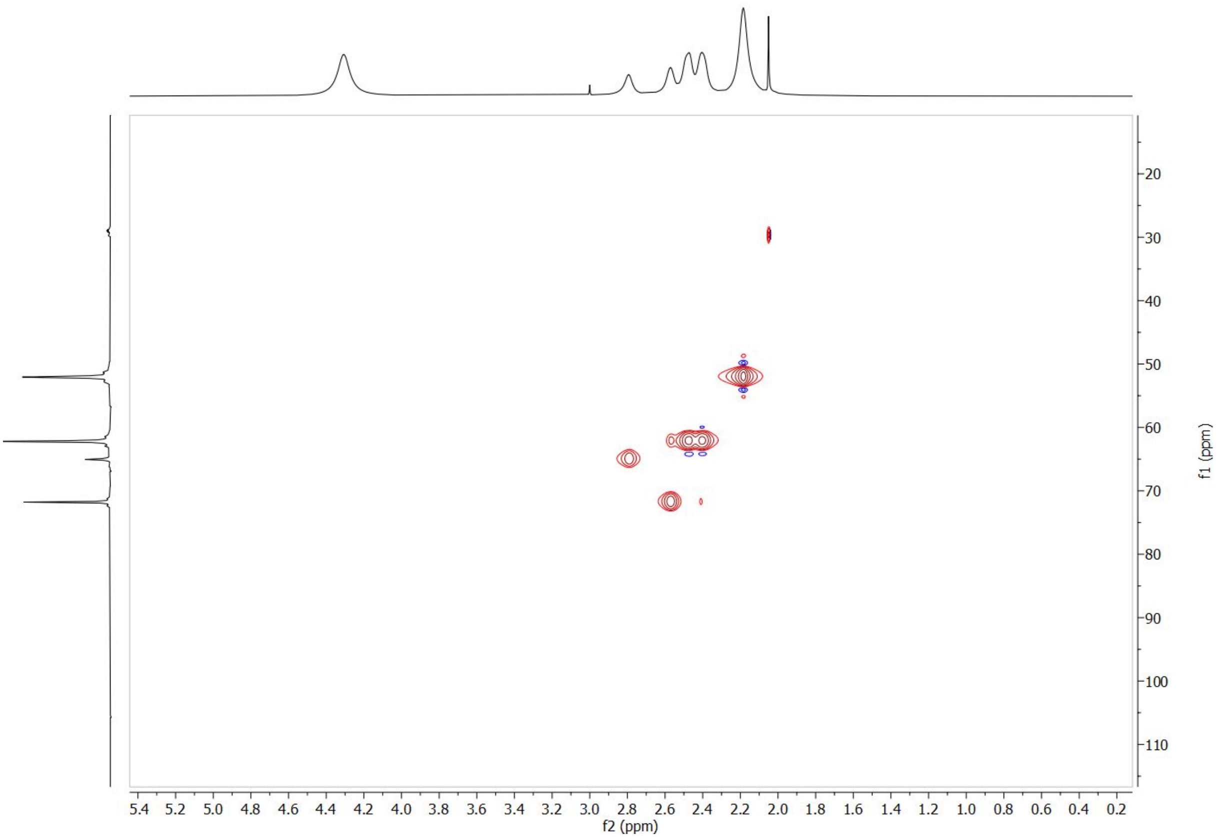

Figure S9. ^1H-^{13}C HSQC spectrum of Bet:Gly.

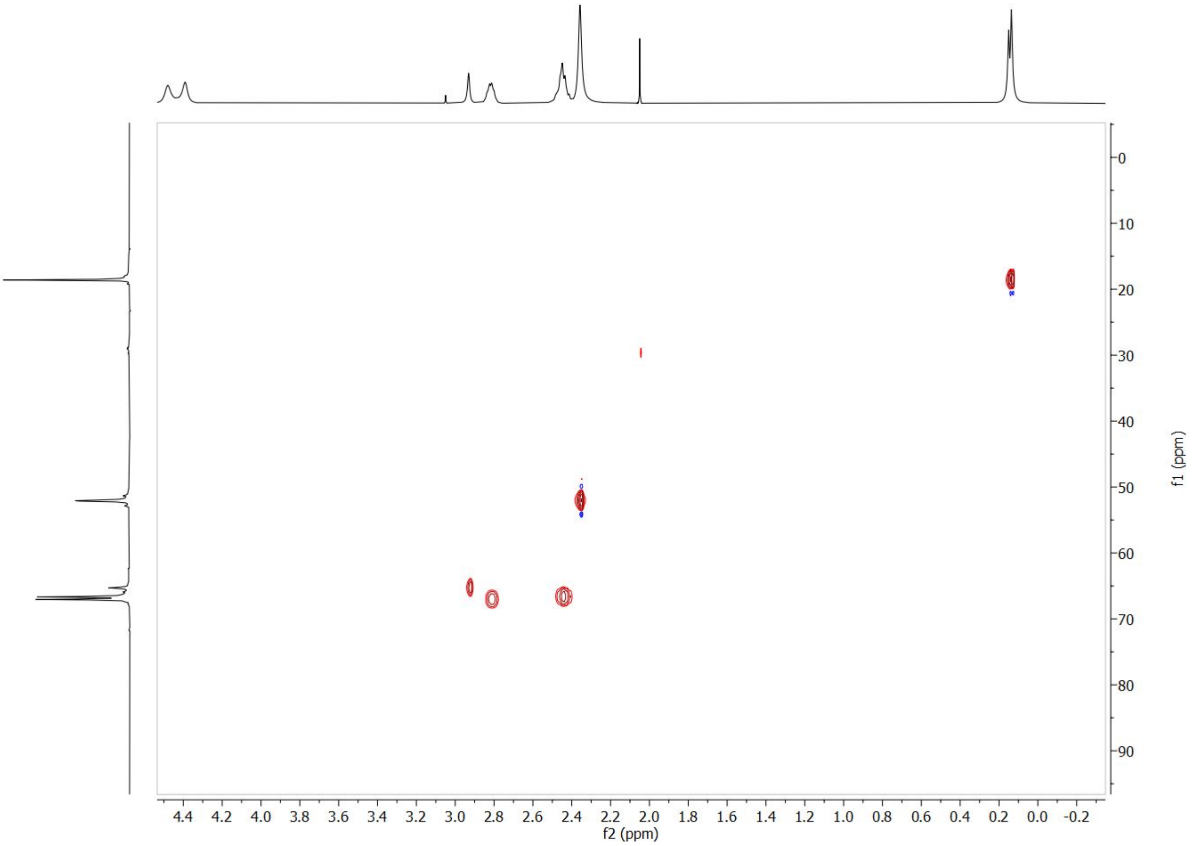

Figure S10. ^1H-^{13}C HSQC spectrum of Bet:PG.

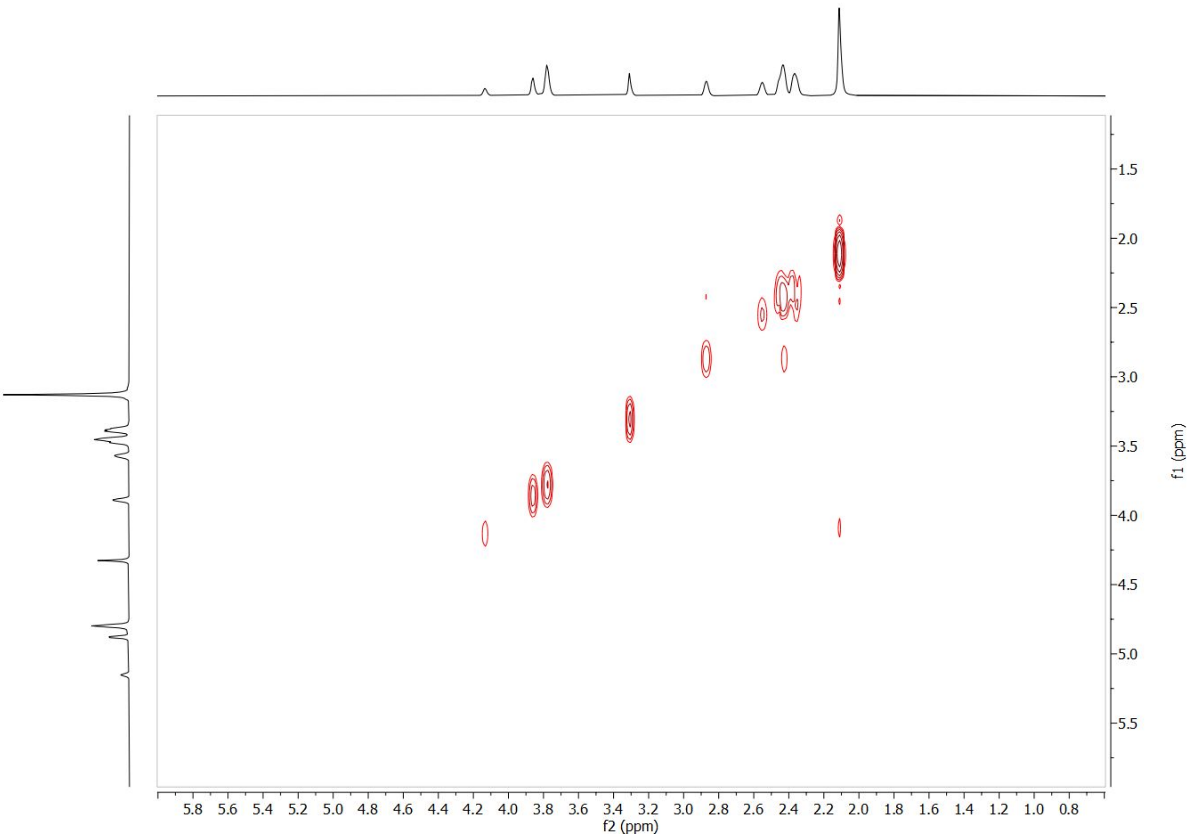

Figure S11. ^{1}H-^{1}H COSY spectrum of ChCl:Gly.

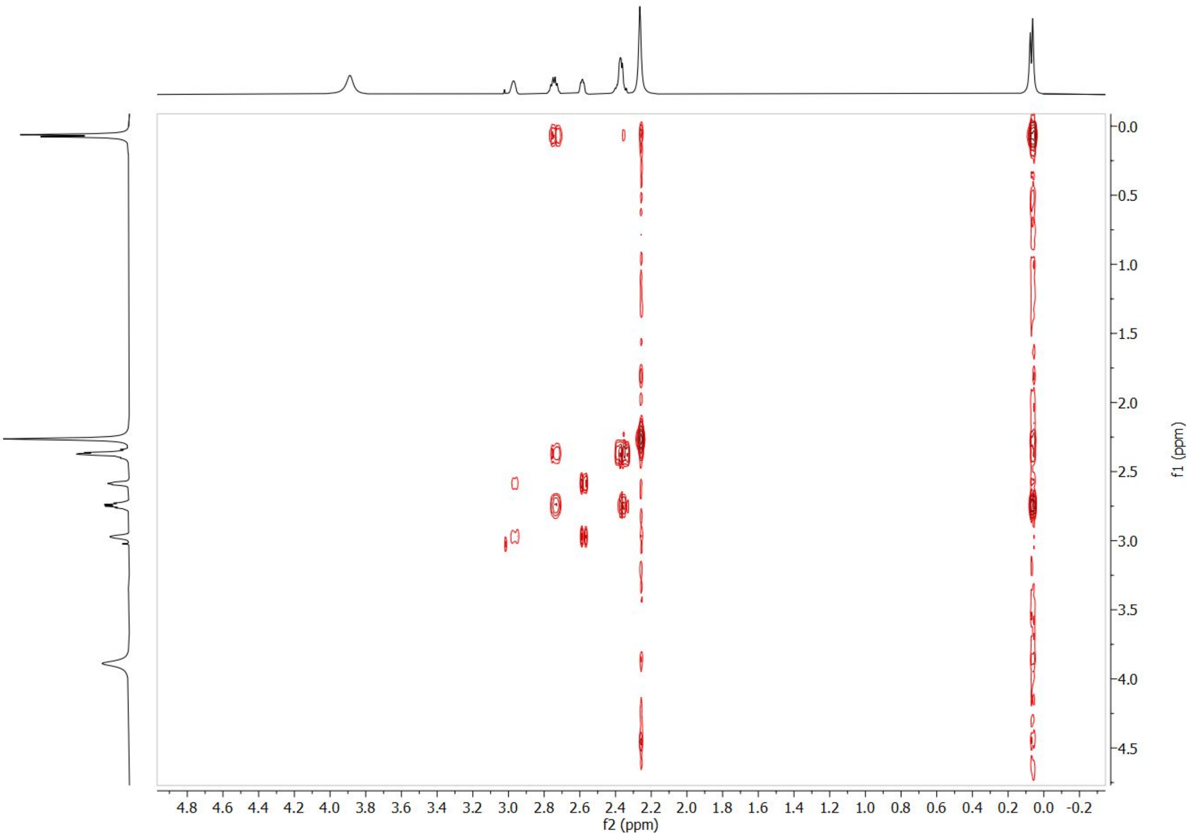

Figure S12. ^{1}H-^{1}H COSY spectrum of ChCl:PG.

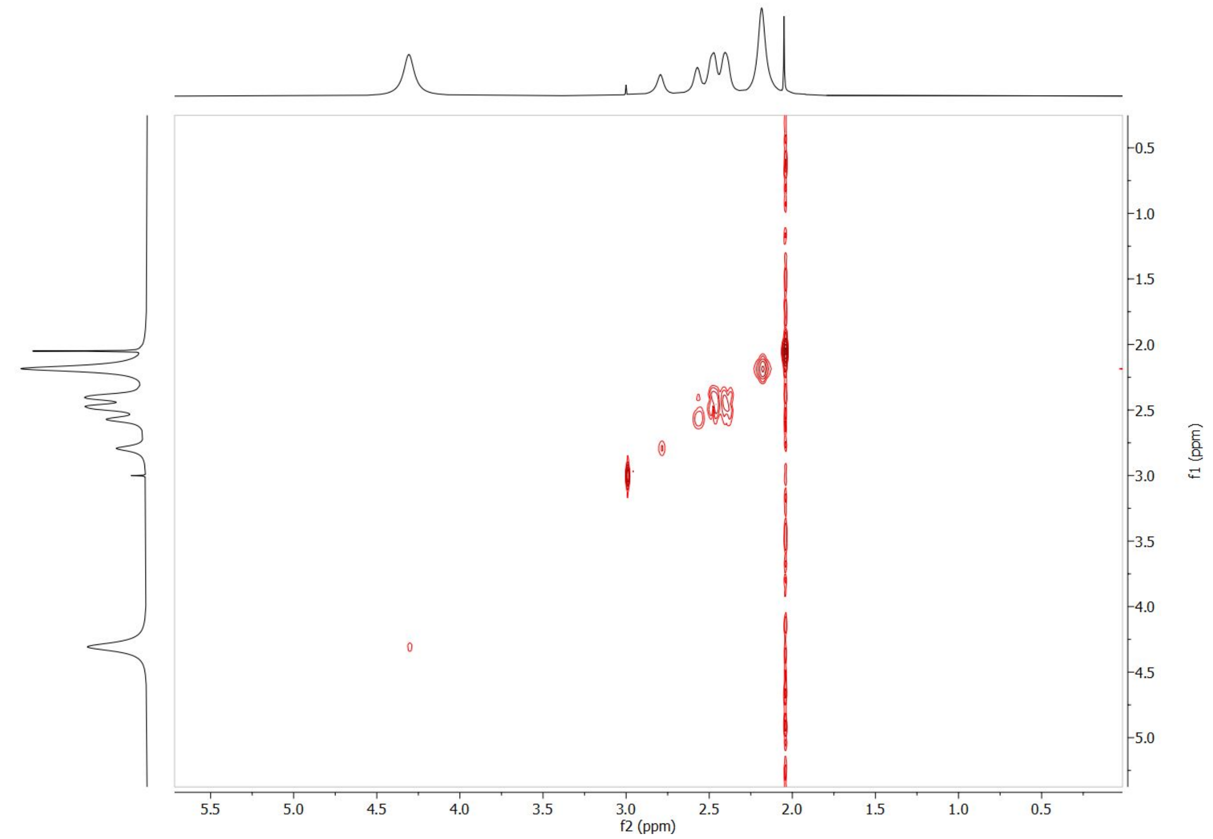

Figure S13. ^{1}H-^{1}H COSY spectrum of Bet:Gly.

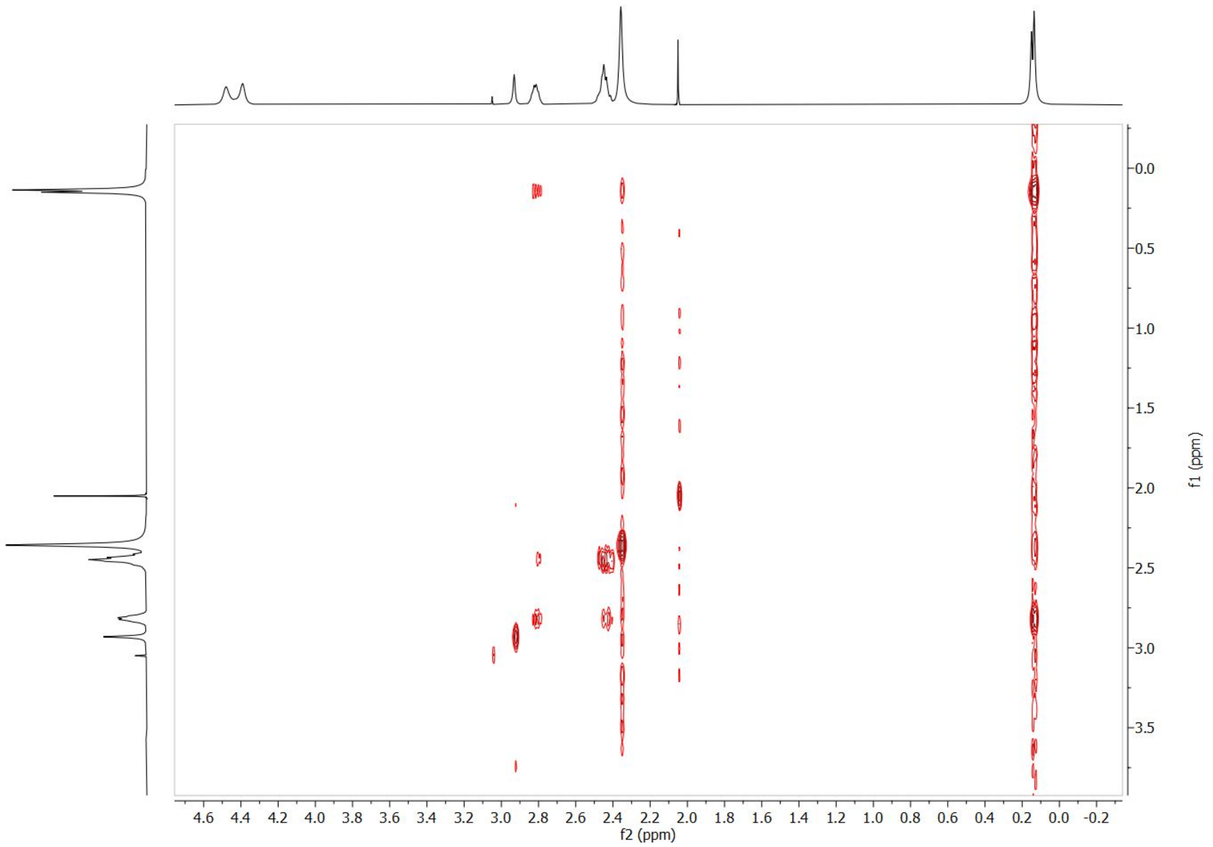

Figure S14. ^{1}H-^{1}H COSY spectrum of Bet:PG.

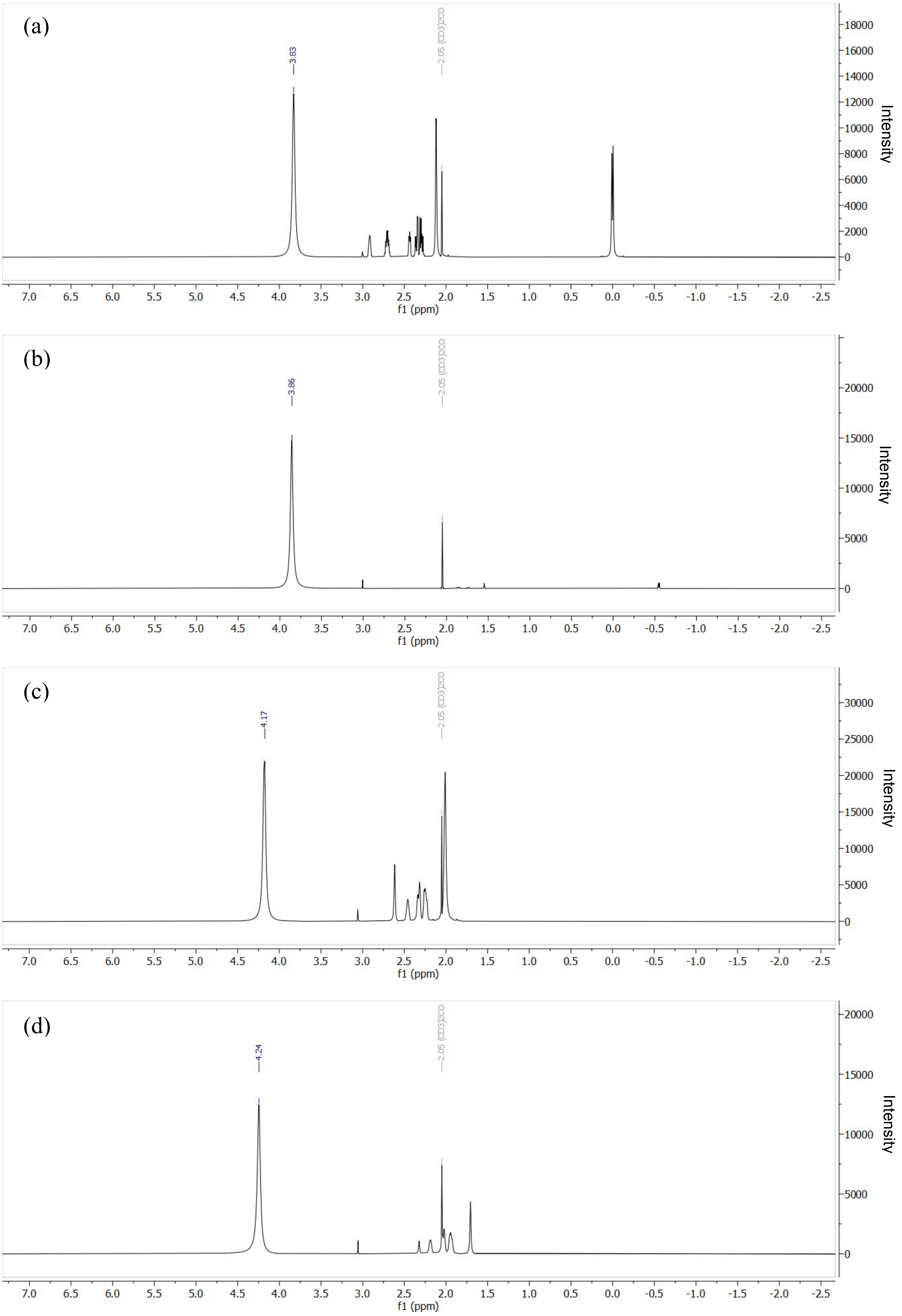
(a)
3.83
2.05 (CD3)2CO
Intensity
f1 (ppm)
(b)
3.86
2.05 (CD3)2CO
Intensity
f1 (ppm)
(c)
4.17
2.05 (CD3)2CO
Intensity
f1 (ppm)
(d)
4.24
2.05 (CD3)2CO
Intensity
f1 (ppm)

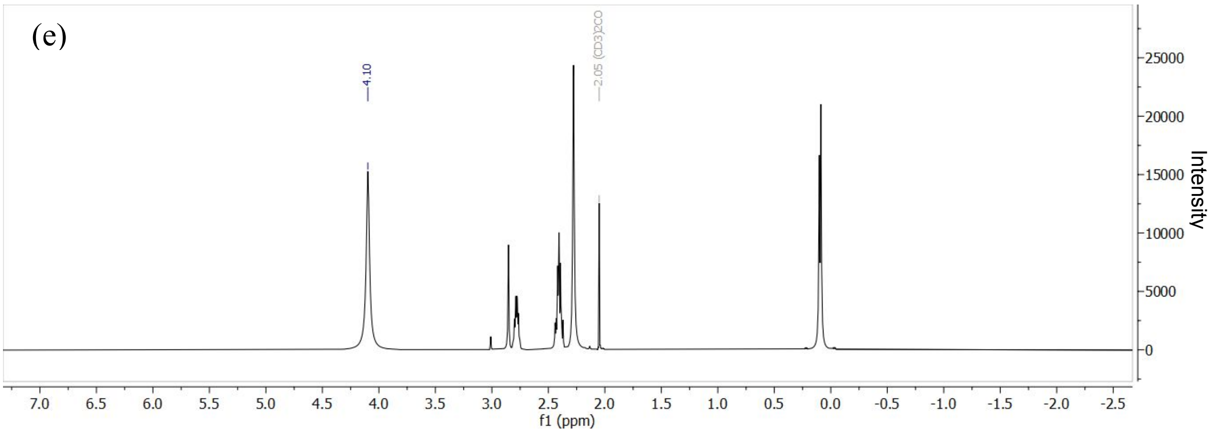

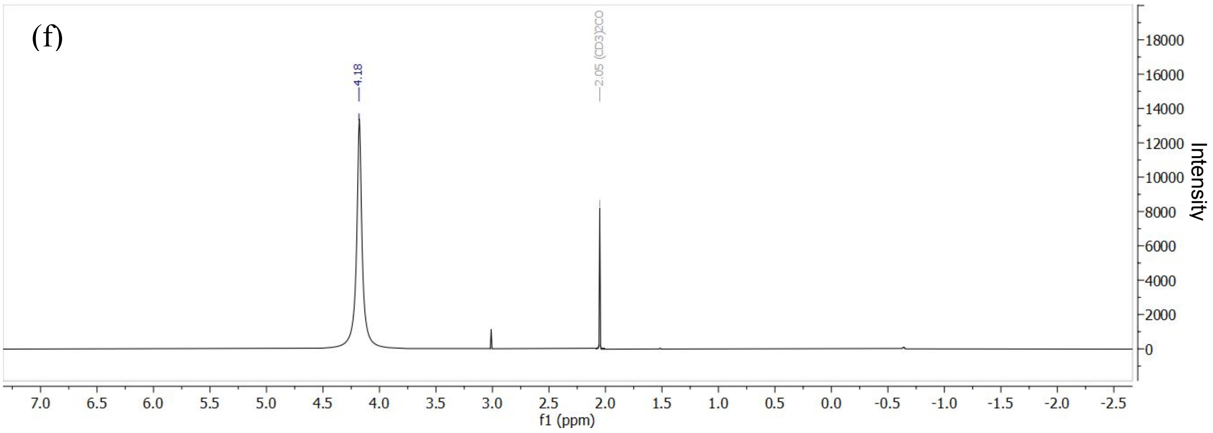

Figure S15. The ^{1}H NMR spectra of (a) the top phase of ChCl:PG-K_2HPO_4 ATPS, (b) the bottom phase of ChCl:PG-K_2HPO_4 ATPS, (c) the top phase of Bet:Gly-K_2HPO_4 ATPS, (d) the bottom phase of Bet:Gly-K_2HPO_4 ATPS, (e) the top phase of Bet:PG-K_2HPO_4 ATPS, and (f) the bottom phase of Bet:PG-K_2HPO_4 ATPS.
